# Glucose homeostasis controls N-acetyltransferase 10-mediated ac4C modification of HK2 to drive gastric tumorigenesis

**DOI:** 10.7150/thno.104310

**Published:** 2025-01-20

**Authors:** Qiang Wang, Mengmeng Li, Chen Chen, Lei Xu, Yao Fu, Jiawen Xu, Chuanjun Shu, Bo Wang, Zhangding Wang, Changyu Chen, Tao Song, Shouyu Wang

**Affiliations:** 1 Department of Hepatobiliary Surgery, The First Affiliated Hospital of Anhui Medical University; MOE Innovation Center for Basic Research in Tumor Immunotherapy; Anhui Province Key Laboratory of Tumor Immune Microenvironment and Immunotherapy, Hefei, China.; 2 Jiangsu Key Laboratory of Molecular Medicine, Medical School of Nanjing University, Nanjing, China.; 3 Department of Gastroenterology, The Affiliated Drum Tower Hospital of Nanjing University Medical School, Nanjing, China.; 4 Department of Pathology, The Affiliated Drum Tower Hospital of Nanjing University Medical School, Nanjing, China.; 5 Department of Bioinformatics, School of Biomedical Engineering and Informatics, Nanjing Medical University, Nanjing, China.; 6 Department of General Surgery, The First Affiliated Hospital of Anhui Medical University, Hefei, China.; 7 Department of Gastrointestinal Surgery, Xuzhou Central Hospital, Xuzhou, China.

**Keywords:** glycolysis, NAT10, ac4C, HK2, gastric cancer

## Abstract

**Rationale:** Abnormal metabolic states contribute to a variety of diseases, including cancer. RNA modifications have diverse biological functions and are implicated in cancer development, including gastric cancer (GC). However, the direct relationship between glucose homeostasis and 4-acetylcytosine (ac4C) modification in GC remains unclear.

**Methods:** The prognostic value of RNA acetyltransferase NAT10 expression was evaluated in a human GC cohort. Additionally, preoperative PET/CT data from GC patients and Micro-PET/CT imaging of mice were employed to assess the relationship between NAT10 and glucose metabolism. The biological role of NAT10 in GC was investigated through various experiments, including GC xenografts, organoids, and a conditional knockout (cKO) mouse model. The underlying mechanisms were examined using dot blotting, immunofluorescence staining, co-immunoprecipitation, and high-throughput sequencing, among other techniques.

**Results:** Glucose deprivation activates the autophagy-lysosome pathway, leading to the degradation of NAT10 by enhancing its interaction with the sequestosome 1 (SQSTM1)/microtubule-associated protein 1 light chain 3 alpha (LC3) complex, ultimately resulting in a reduction of ac4C modification. Furthermore, the levels of ac4C and NAT10 are elevated in GC tissues and correlate with poor prognosis. A strong correlation exists between NAT10 levels and 18F-FDG uptake in GC patients. Furthermore, NAT10 drives glycolytic metabolism and gastric carcinogenesis *in vitro* and* in vivo*. Mechanistically, NAT10 stimulates ac4C modification at the intersection of the coding sequence (CDS) and 3' untranslated region (3'UTR) of hexokinase 2 (HK2) mRNA, enhancing its stability and activating the glycolytic pathway, thereby driving gastric tumorigenesis.

**Conclusion:** Our findings highlight the critical crosstalk between glucose homeostasis and the ac4C epitranscriptome in gastric carcinogenesis. This finding offers a potential strategy of targeting NAT10/HK2 axis for the treatment of GC patients, especially those with highly active glucose metabolism.

## Introduction

As the fifth most common malignancy and the fourth leading cause of cancer-related death globally 1, gastric cancer (GC) is a major cause of morbidity and mortality worldwide. Although early endoscopic diagnosis and treatment with surgery, chemoradiotherapy, targeted treatment and other comprehensive treatment programs has led to a decreasing trend in the incidence and mortality of GC 2-4, the overall prognosis is still poor. Therefore, identifying novel biomarkers and potential therapeutic targets for GC is urgently needed.

Reprogramming of energy metabolism is one of the distinct hallmarks of cancer and is also a focus in the development of anticancer drugs 5, 6. The Warburg effect, i.e., active glycolysis and diminished mitochondrial aerobic metabolism, is considered one of the main characteristics of tumor metabolic reprogramming 6-8. Our previous study reported that N6-methyladenosine (m6A) RNA methylation-mediated glycolysis is a key process involved in GC progression 9. Targeting players in aerobic glycolysis, including glucose transporters, glycolytic enzymes, and related pathways, is an attractive therapeutic intervention, and several inhibitors specifically targeting aerobic glycolysis have shown potential therapeutic efficacy in preclinical studies 7, 10, 11. Thus, understanding the molecular mechanisms of glycolysis in GC is essential for developing diagnostic and therapeutic strategies.

More than 170 types of RNA chemical modifications have been identified, among which are various types of modifications on transport RNAs (tRNAs) and ribosomal RNAs (rRNAs)^12^, ^13^. In recent years, various modifications, such as 6-methyladenine (m6A), 4-acetylcytosine (ac4C), 5-methylcytosine (m5C), 7-methylguanine (m7G), and 1-methyladenine (m1A), have been found on mRNAs and noncoding RNAs 12, 14, 15. These dynamic and reversible chemical modifications have diverse biological functions and are involved in the development of various diseases, including cancer 14, 16, 17.

Recent studies on the functional mechanism of m6A modification have been conducted in depth 18, 19. Our group has conducted a series of studies on the role of m6A modification in various tumors 9, 20-24. At the end of 2018, a study revealed mRNA ac4C modification for the first time. It was found that ac4C modification was enriched mainly in the coding region (CDS) and 5' untranslated region (5'-UTR), and N-acetyltransferase 10 (NAT10) is currently the only RNA acetyltransferase that has been found to catalyse ac4C modification to maintain mRNA stability and enhance translation efficiency 25. Recent studies have revealed that NAT10-mediated ac4C modification can mediate growth and metastasis in different cancers, including GC 26-31; however, the relationship between glucose homeostasis and ac4C modification in tumorigenesis has not been fully elucidated.

In the present study, we identified the crosstalk between glucose metabolism and ac4C modification-driven gastric tumorigenesis. Glucose deprivation activated autophagy to promote the interaction between NAT10 and the sequestosome 1 (SQSTM1)/microtubule-associated protein 1 light chain 3 alpha (LC3) complex, leading to degradation of NAT10 through the lysosomal pathway. On the other hand, NAT10 catalysed ac4C acetylation of hexokinase 2 (HK2) mRNA and promoted aerobic glycolysis and gastric tumorigenesis. These findings revealed critical crosstalk between glucose homeostasis and the ac4C epitranscriptome in gastric carcinogenesis.

## Results

### Glucose deprivation activates the autophagosomal pathway to degrade NAT10, leading to a decrease in ac4C abundance

**Figure 1:**
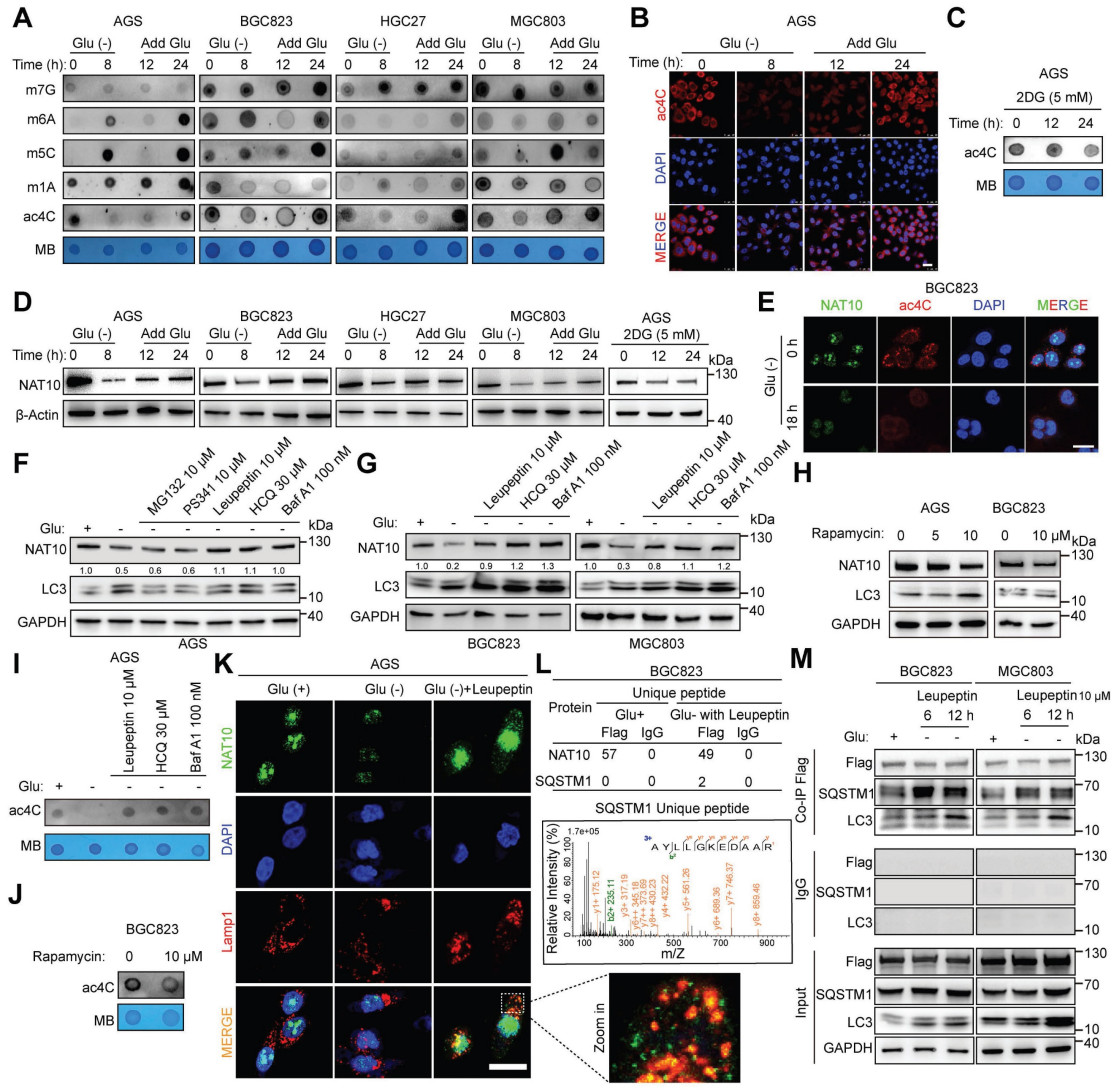
** Glucose status controls NAT10-mediated ac4C modification. (A)** mRNA was isolated from four different GC cell lines (AGS, BGC823, HGC27, and MGC803), which were subjected to glucose starvation for the indicated intervals and then stimulated with glucose (25 mM) for the indicated intervals. The mRNAs were subjected to dot blot analysis with the indicated antibodies (specific for m7G, m6A, m5C, m1A, and ac4C), and MB (methylene blue) staining was used as the loading control. **(B)** The localization and abundance of ac4C (red) were evaluated by immunofluorescence staining in AGS cells treated with or without glucose for the indicated times (scale bars = 25 μm). **(C)** Dot blot analysis was used to measure the ac4C abundance after treatment with an anti-ac4C antibody in AGS cells treated with 5 mM 2DG for 0, 12 and 24 h. **(D)** Different GC cell lines (AGS, BGC823, HGC27, and MGC803) were subjected to glucose starvation for the indicated intervals and then stimulated with glucose (25 mM) for the indicated intervals (left panel); alternatively, AGS cells were treated with 5 mM 2DG for 0, 12 and 24 h. Cell lysates obtained at each time point were subjected to Western blot analysis. **(E)** Confocal images showing the localization and levels of ac4C (red) and NAT10 (green) in BGC823 cells cultured in the presence or absence of glucose (scale bars = 25 μm). **(F)** Proteasome inhibitors (MG132 and PS341), a lysosome inhibitor (leupeptin) and autophagy inhibitors (HCQ and Baf A1) were added to glucose-deprived AGS cells for 8 h. Then, the cell lysates were subjected to Western blot analysis. **(G)** A lysosome inhibitor (leupeptin) and autophagy inhibitors (HCQ, Baf A1) were added to glucose-deprived GC cells (BGC823 and MGC803) for 8 h. Then, the cell lysates were subjected to Western blot analysis. **(H)** The autophagy agonist rapamycin was added at the indicated concentration to glucose-deprived GC cells (AGS and MGC803) for 24 h. Then, the cell lysates were subjected to Western blot analysis. **(I)** A lysosome inhibitor (leupeptin) and autophagy inhibitors (HCQ, Baf A1) were added to glucose-deprived GC cells (AGS) for 8 h. Then, mRNA was isolated for use in a dot blot assay. **(J)** The autophagy agonist rapamycin was added at the indicated concentration to glucose-deprived GC cells (BGC823) for 24 h. Then, mRNA was isolated for use in a dot blot assay. **(K)** Confocal images showing the localization and levels of Lamp1 (red) and NAT10 (green) in AGS cells cultured in three types of glucose-containing media (regular glucose-rich medium (25 mM glucose), glucose-free medium, or glucose-free medium containing the lysosome inhibitor leupeptin (scale bars = 25 μm). **(L)** The proteins that interact with NAT10 were identified by IP-MS, and the specific peptide (AYLLGKEDAAR) of SQSTM1/p62 was detected in the GC cell line (BGC823) cultured in glucose-free medium containing leupeptin.** (M)** The GC cell lines (BGC823 and MGC803) were cultured in two types of glucose-containing media (regular glucose-rich medium (25 mM glucose), or glucose-free medium containing the lysosome inhibitor leupeptin) for 6 or 12 h, and cell lysates from the different groups were subjected to co-IP assays. 2DG at 5 mM, MG132 at 10 μM, PS341 at 10 μM, leupeptin at 10 μM, HCQ at 30 μM, Baf A1 at 100 nM, and rapamycin at 10 μM were used in the above experiments. Glu, glucose. The data in this figure were repeated in at least three independent experiments.

To explore whether glucose status affects the abundance of different RNA modifications, we first examined the m7G, m6A, m5C, m1A, and ac4C levels in GC cells with different glucose status (Figure 1A). We found that glucose deprivation consistently decreased the abundance of ac4C; the ac4C abundance increased when glucose was added back to glucose-deprived cells, while the changes in other RNA modifications were inconsistent in the four different GC cell lines (Figure 1A and S1A). Therefore, we focused on the role of glucose status in the regulation of ac4C modification. An immunofluorescence (IF) assay confirmed that glucose status controlled the ac4C modification abundance (Figure 1B). To further validate these findings, an inhibitor of the glycolytic pathway (2-deoxy-D-glucose, 2-DG), which is a glucose molecule in which the 2-hydroxyl group is replaced by hydrogen to induce cellular energy stress, was used 32. The abundance of ac4C was decreased in GC cells treated with 2-DG (Figure 1C). These data suggest that glucose status regulates the abundance of ac4C.

Next, we investigated whether the ac4C abundance is modulated by NAT10 after glucose stress. Notably, glucose starvation strongly decreased the NAT10 protein abundance, which increased when glucose was added back to glucose-deprived cells in the four tested human GC cell lines (Figure 1D). However, the NAT10 mRNA level did not change consistently in the four GC cell lines (Figure S1B). Moreover, 2-DG treatment resulted in a decrease in the protein abundance but not the mRNA level of NAT10 (Figure 1D and S1C). Furthermore, we observed that the NAT10 level in the nucleus decreased upon glucose deprivation, and this change was accompanied by a decrease in the ac4C abundance (Figure 1E). These results suggest that glucose deprivation may reduce NAT10-mediated ac4C abundance through posttranslational regulation.

A previous study indicates that autophagy can be initiated in response to stimuli such as metabolic stress and glucose deprivation 33. Glucose deprivation activates autophagy through multiple pathways, including the AMPK-mediated energy stress response, inhibition of mTORC1, mitochondrial dysfunction, production of reactive oxygen species (ROS), and activation of transcription factors. These mechanisms work together to help cells cope with energy deficiency, maintaining cell survival and function 34, 35. Our results also revealed an increase in the ratio of LC3-II to LC3-I, a hallmark of autophagy 33, 36, in response to glucose deprivation in different GC cell lines (Figure 1F and 1G), indicating that autophagy was activated in response to glucose deprivation in GC cells. To explore how glucose deprivation mediates the degradation of the NAT10 protein, ubiquitin‒proteasome inhibitors (MG132, PS341)^37^, a lysosome inhibitor (leupeptin)^38^ and autophagy inhibitors (HCQ, Baf A1)^38^ were added to glucose-deprived GC cells. Notably, only the lysosome/autophagy inhibitor rescued the degradation of NAT10 in response to glucose deprivation (Figure 1F and 1G). Moreover, the protein expression of NAT10 in GC cells decreased after treatment with rapamycin, an autophagy activator^38^ (Figure 1H). As expected, the lysosome/autophagy inhibitor rescued the decrease in the ac4C abundance in GC cells under glucose deprivation (Figure 1I), while rapamycin activated autophagy and decreased the ac4C abundance in GC cells (Figure 1J). Furthermore, the IF assay revealed the cytoplasmic colocalization of NAT10 and LAMP1, a lysosomal marker^39^, in glucose-deprived GC cells treated with leupeptin (Figure 1K). To further identify the specific molecules involved in NAT10 degradation via the autophagy/lysosome pathway, immunoprecipitation followed by mass spectrometry (IP-MS) was performed on the GC cell line BGC823 cultured with different concentrations of glucose (25 mM glucose medium, or glucose-free medium containing leupeptin). Notably, SQSTM1/p62, an LC3-binding protein and receptor 36, 40, was found to bind to NAT10 in glucose-free medium containing leupeptin (Figure 1L). Co-IP experiments also showed that the interactions between NAT10 and both SQSTM1/p62 and LC3 were more pronounced in glucose-free medium supplemented with leupeptin (Figure 1M).

**Figure 2:**
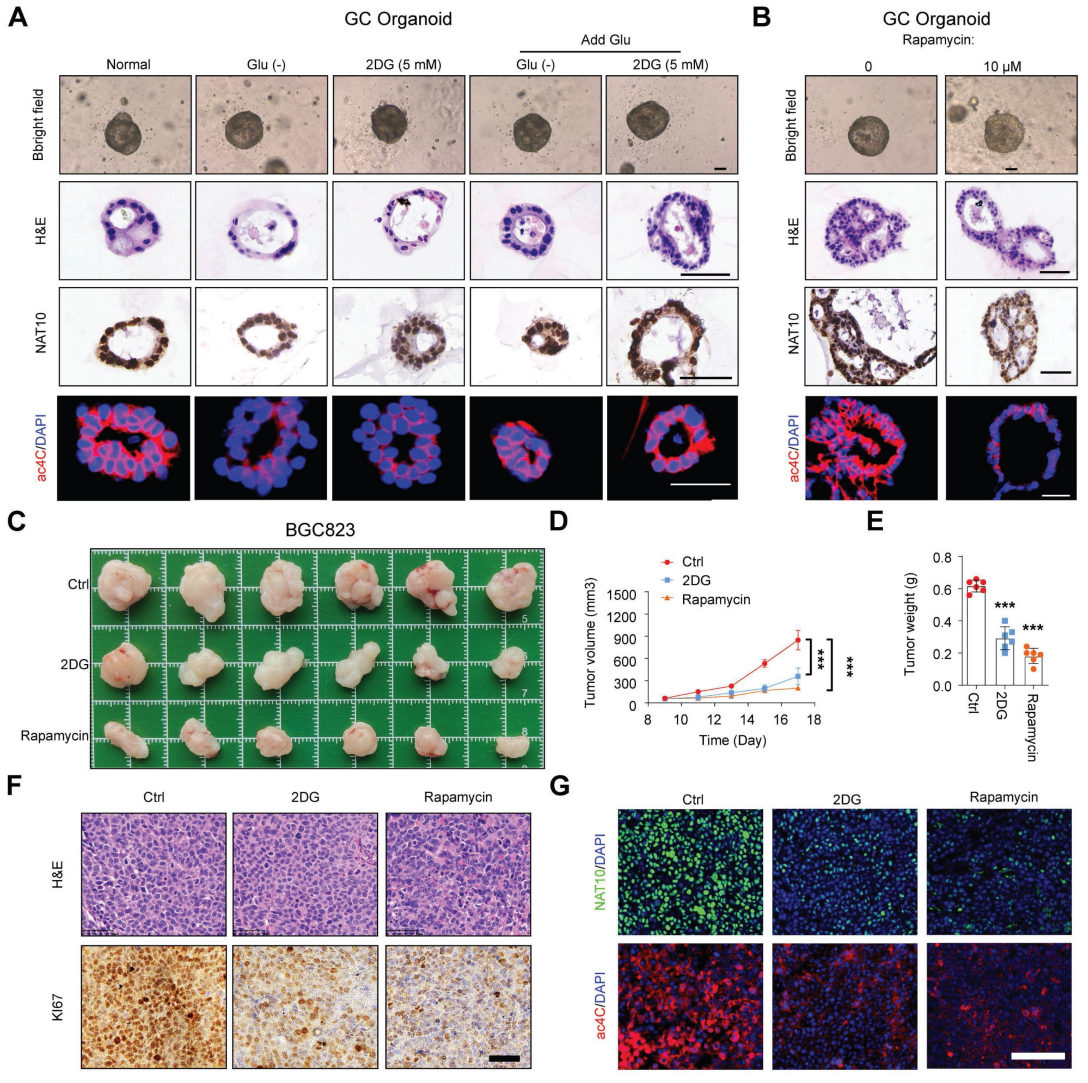
** Glucose status regulates NAT10-mediated ac4C modification. (A, B)** The GC organoids were subjected to glucose starvation or treated with 5 mM 2DG for 12 h, and then stimulated with glucose (25 mM) for additional 24 h **(A)**; alternatively, the GC organoids were treated with autophagy agonist rapamycin at 0 or 10 μM for 24 h **(B)**. Then representative images of GC organoids were showed (scale bars = 50 μm), and the sections of organoids from different groups were subjected to H&E staining or stained with anti-NAT10 antibodies for IHC analysis or stained with anti-ac4C antibodies for IF analysis (scale bars = 50 μm). **(C)** 2DG (800 mg/kg) and Rapamycin (4 mg/kg) were injected intraperitoneally once a day (5 times in total) when the tumor grows to 50-100mm^3^ at Day 9. At Day 18, the mice were sacrificed, and the tumors were weighed and imaged (n = 6). 2-DG or rapamycin treatments both significantly inhibited subcutaneous tumor growth. The tumor volume was monitored every other day, and tumor growth curves were generated **(D)**. The tumors were extracted and weighed after 18 days **(E)**. **(F)** Sections of tumors were for H&E staining or stained with anti-Ki-67 antibodies for IHC analysis (scale bars = 50 μm). **(G)** Sections of tumors were stained with anti-NAT10 or anti-ac4C antibodies for IF analysis (scale bars = 100 μm). Glu, glucose. The data in this figure were repeated three independent experiments. The statistical data in this figure are presented as the mean value ± SD of three independent experiments. Statistical significance was determined by a two-tailed t test. * P < 0.05; ** P < 0.01; *** P < 0.001.

To further explore whether glucose homeostasis regulates NAT10-mediated ac4C modification, we examined the levels of NAT10 and ac4C in GC organoids with different glucose status, which showed that both short-term glucose deprivation or treatment with 2-DG could significantly decrease the levels of NAT10 and ac4C; conversely, these levels increased when glucose was added back to glucose-deprived organoids or those treated with 2-DG (Figure 2A). Additionally, we confirmed that the autophagy activator rapamycin also led to a decrease in NAT10 and ac4C levels in GC organoids (Figure 2B). However, during the short-term models, we did not observe significant morphological changes in GC organoids under conditions of glucose deficiency or autophagy activation (Figure 2A and 2B). To further assess the effect of long-term glucose deficiency or autophagy activation on GC progression, we constructed a GC cell line subcutaneous xenograft model and treated the mice with 2-DG or rapamycin. The *in vivo* results showed that, compared with that in the control group, both 2-DG and rapamycin treatments significantly suppressed tumor growth, as indicated by the decreases in tumor size and weight (Figure 2C, 2D, and 2E). In addition, the IHC results showed decreased expression KI-67 in the 2-DG and rapamycin treatment groups compared to the control group (Figure 2F). Furthermore, we found both the levels of NAT10 protein and ac4C were markedly decreased in the 2-DG and rapamycin treatment groups compared to the control group (Figure 2G), while NAT10 mRNA levels did not exhibit consistent changes (Figure S1D). Taken together, our findings suggested that glucose deprivation could activate autophagy to promote the interaction between NAT10 and SQSTM1/LC3, causing NAT10 to be delivered into lysosomes for degradation. And this leads to a decrease in ac4C abundance in glucose deprivation.

### Elevated NAT10 expression is correlated with poor prognosis in patients with GC

**Figure 3:**
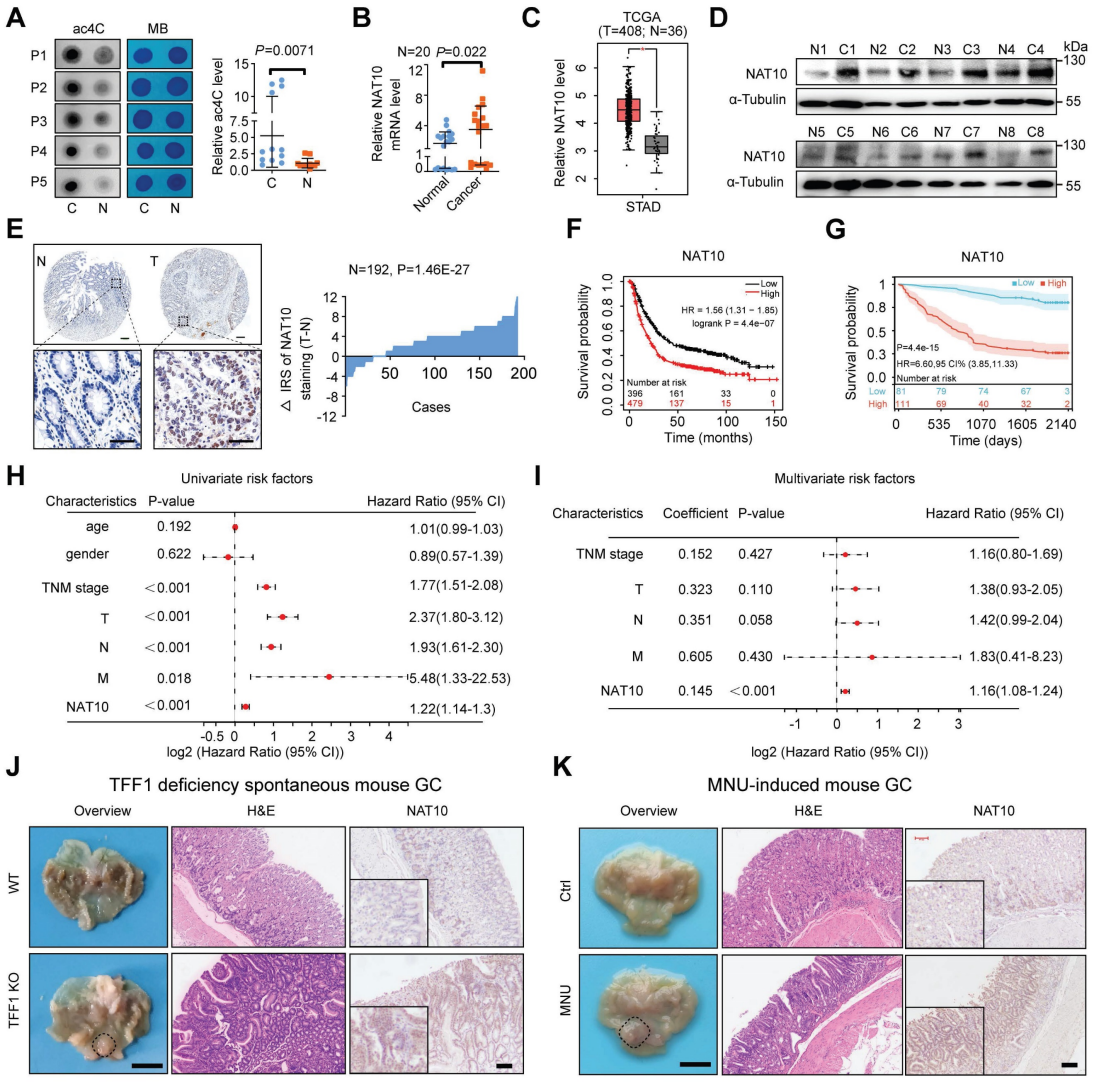
** Elevated NAT10 expression correlates with poor prognosis in GC patients. (A)** mRNA isolated from GC tissues and paired normal gastric mucosa was subjected to dot blot analysis with an anti-ac4C antibody, and MB staining served as the loading control (representative images in the left panel). The relative ac4C abundance on mRNA in GC tissues and paired normal gastric mucosal tissues was calculated (right panel, n = 12). **(B)** The level of NAT10 expression in GC and paired normal gastric mucosal tissues was measured by qRT-PCR (n = 20).** (C)** The level of NAT10 expression was analysed in GC (n = 408) and normal gastric mucosal tissues (n = 36) using TCGA data. **(D)** The NAT10 protein level was measured in GC tissues and paired normal gastric mucosal tissues by Western blotting (n = 8). **(E)** Representative IHC images of the tissue microarray analysed with the anti-NAT10 antibody (scale bars = 200 or 100 μm) are shown (left panel). The distribution of the difference in the NAT10 immunoreactivity score (IRS) (△IRS = IRST - IRSN) is shown. The IRS for NAT10 staining was available for 192 pairs of tissues.** (F)** Kaplan-Meier curves of overall survival (OS) based on NAT10 expression were generated using the online bioinformatics tool Kaplan-Meier Plotter. **(G)** Kaplan-Meier analysis of OS in GC patients stratified by NAT10 expression (n = 192, P = 4.4e-15; log-rank test).** (H)** Univariate analyses were performed in the GC cohort.** (I)** Multivariate analyses were performed in the GC cohort. **(J)** Representative macroscopic images (scale bars = 0.5 cm), H&E staining images (scale bars = 100 μm), and NAT10 IHC staining images (scale bars = 100 μm) of stomachs from 8-month-old TFF1 KO mice and WT mice. **(K)** Representative macroscopic images (scale bars = 0.5 cm), H&E staining images (scale bars = 100 μm), and NAT10 IHC staining images (scale bars = 100 μm) of stomachs from 35-week-old WT mice and mice with MNU-induced GC. All bars correspond to 95% confidence intervals (CIs). HR: hazard ratio; H&E, haematoxylin and eosin; GC, gastric cancer; MNU, N-methyl-N-nitrosourea; OS, overall survival; TNM, tumor, node, metastasis; WT, wild type. The statistical data in this figure are presented as the mean value ± SD of three independent experiments. Statistical significance was determined by a two-tailed t test. * P < 0.05; ** P < 0.01; *** P < 0.001.

It was demonstrated in our previous study that the abundance of RNA m6A modifications was higher in cancerous tissues than in paired normal gastric mucosa 9, 24. Here, we examined other RNA modifications, such as m7G, m5C, m1A, and ac4C, in GC tissues. Via a dot blot assay, we found that the RNA ac4C abundance was significantly higher in GC tumor tissues (Figure 3A), while the abundances of other RNA modifications (m7G, m5C, m1A) were not significantly different (Figure S2A). Moreover, the NAT10 mRNA level was significantly increased in GC tumor tissues (Figure 3B). Furthermore, analysis of data from The Cancer Genome Atlas (TCGA) confirmed that NAT10 was significantly upregulated in diverse types of tumor tissues, including GC (Figure 3C and S2B). Consistently, the protein level of NAT10 was found to be significantly higher in 8 cancerous tissues than in the paired normal gastric tissues by Western blot analysis (Figure 3D). These results were confirmed by immunohistochemistry (IHC) staining of a tissue microarray (TMA) containing samples from patients with GC, which revealed that the expression of NAT10 was significantly increased in GC tissues compared with the matched normal gastric tissues (n = 192, P = 1.46E-27; Figure 3E). Using the online bioinformatics tool Kaplan‒Meier Plotter (http://kmplot.com/analysis/), we also found that GC patients with increased NAT10 mRNA levels had worse overall survival (OS) (Figure 3F). Consistently, data from our GC cohort also showed that GC patients with increased NAT10 protein expression had worse OS (n = 192, P = 4.4E-15; log-rank test; Figure 3G). Furthermore, in GC patients, univariate Cox regression analysis revealed strong associations between survival and TNM stage; individual tumor (T), node (N), and metastasis (M) classifications; and NAT10 expression (Figure 3H). Multivariate Cox regression analysis indicated that NAT10 expression was an independent predictor of prognosis in patients with GC (Figure 3I). It is widely known that TFF1 protein deficiency in mice can lead to spontaneous GC and that N-methyl-N-nitrosourea (MNU) can induce gastric tumorigenesis in mice 41, 42. Our results showed that the level of NAT10 in mouse GC tissues was significantly increased compared with that in normal gastric tissues (Figure 3J, 3K, S2C, S2D, and S2E). Taken together, these data reveal that the levels of NAT10 and RNA ac4C modification are increased in GC and that NAT10 might be an independent prognostic factor in GC. Our above results indicated that glucose status controls the NAT10 protein level, prompting our interest to explore whether NAT10 could regulate glucose metabolism in GC via a feedback mechanism.

### NAT10 drives glycolytic metabolism in GC

**Figure 4:**
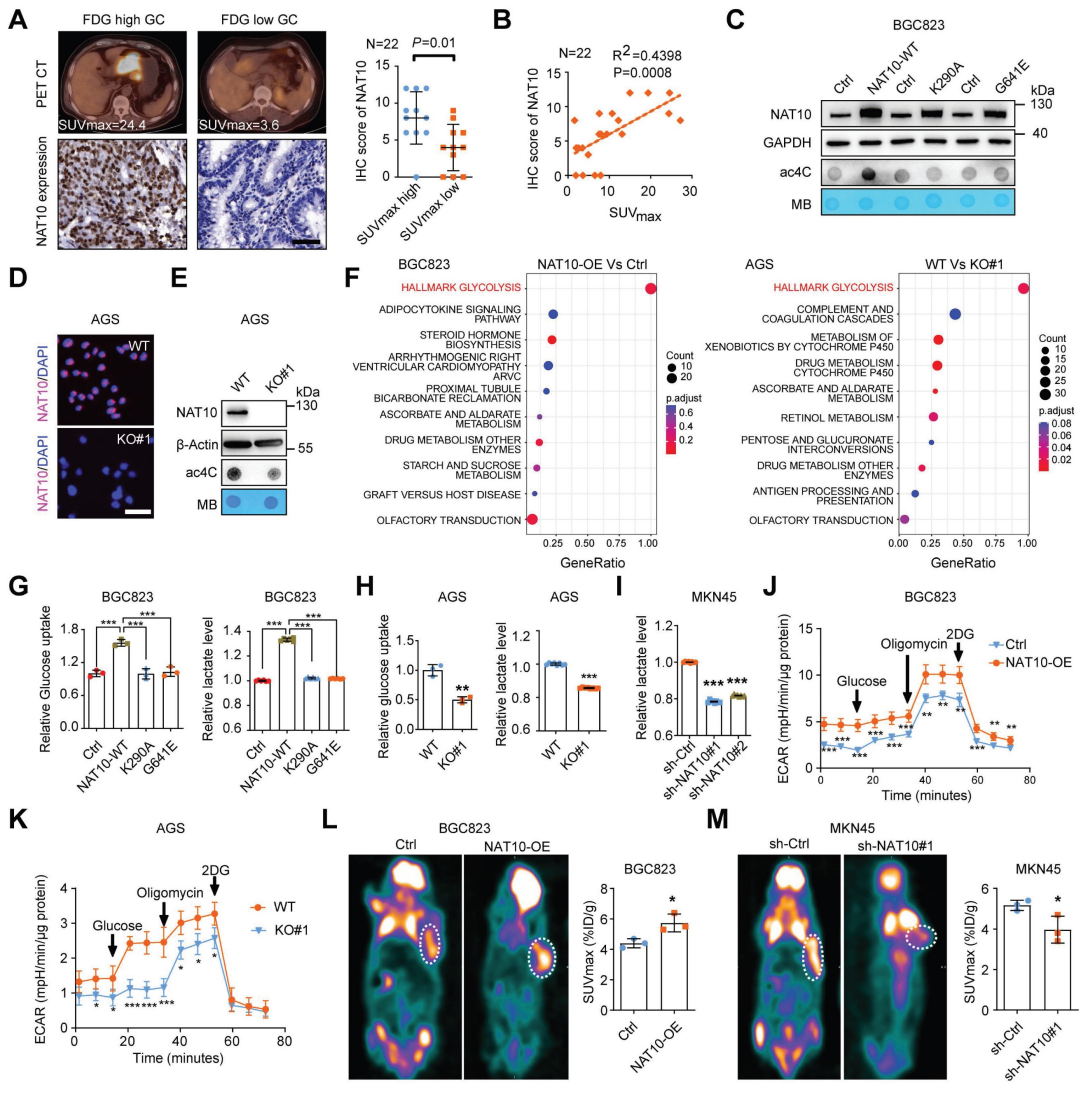
** NAT10 drives glycolytic metabolism in GC. (A)** Representative of PET/CT images and NAT10 IHC staining images from patients with GC (left panel). Scale bars = 50 μm; n = 22; statistical analysis of the difference in NAT10 expression between the SUVmax-high group and the SUVmax-low group (right panel). **(B)** Correlation analysis between the SUVmax and NAT10 protein expression level based on the IHC score in GC tissue microarrays with associated PET/CT data. R, Pearson correlation coefficient. n = 22. **(C)** The protein level of NAT10 in BGC823 cells with overexpression of wild-type or catalytically dead mutant NAT10 were measured by Western blotting (upper panel). mRNA isolated from wild-type or catalytically dead mutant NAT10-overexpressing GC cells were subjected to dot blot analysis with an anti-ac4C antibody (bottom panel). MB staining served as a loading control. **(D)** The protein level of NAT10 in AGS cells with NAT10 knockout was evaluated via IF staining (scale bars = 50 μm). **(E)** The protein level of NAT10 in AGS cells with NAT10 knockout was measured by Western blot analysis (upper panel). mRNA isolated from NAT10-knockout AGS cells were subjected to dot blot analysis with an anti-ac4C antibody (bottom panel). MB staining served as a loading control. **(F)** Pathway analysis indicated that different metabolism-related pathways, especially glycolytic pathways, are involved in GC progression upon upregulation or knockdown of NAT10.** (G)** Glucose uptake (left panel) and lactate production (right panel) in BGC823 cells overexpressing wild-type or catalytically dead mutant NAT10 were determined.** (H)** Glucose uptake (left panel) and lactate production (right panel) in AGS WT and NAT10 KO cells were measured. **(I)** Lactate production in MKN45 cells with NAT10 knockdown was measured. **(J, K)** The ECAR profile was monitored in NAT10-overexpressing **(J)** and NAT10 knockout **(K)** GC cells with a Seahorse XF24 analyser for 100 min. Metabolic inhibitors were injected sequentially at different time points as indicated. **(L, M)** Representative images of 18F-FDG uptake by micro-PET imaging in xenograft mouse models established with NAT10-overexpressing **(L)** or NAT10-knockdown** (M)** cells. The white circles indicate tumor glucose uptake. The maximum standardized uptake values (SUVmax) for xenografts measured by 18F-FDG-PET/CT were determined and quantitatively analysed (n = 3 mice). GC, gastric cancer; WT, wild type; KO, knockout. The statistical data in this figure are presented as the mean ± SD of three independent experiments. Statistical significance was determined by a two-tailed t test. * P < 0.05; ** P < 0.01; *** P < 0.001.

To further explore the correlation between NAT10 expression and glucose metabolism in GC, we first evaluated the expression of NAT10 in 22 GC patients who had undergone [18F]-fluoro-2-deoxyglucose positron emission tomography and computed tomography (18F-FDG PET/CT) with measurement of the FDG maximum standardized uptake value (SUVmax) (Figure 4A). NAT10 expression was significantly increased in the group with a higher SUVmax and was also correlated with FDG uptake in GC patients (Figure 4A and 4B). To further examine the effect of NAT10 expression on glucose metabolism in GC cells, we first measured the level of NAT10 in different GC cell lines, and the results revealed that the mRNA and protein levels of NAT10 were significantly increased in GC cells compared with normal human gastric mucosal tissues (Figure S3A and S3B). Next, GC cells (BGC823 and MGC803) with stable overexpression of wild-type (WT) NAT10 or a catalytically dead mutant (G641E or K290A) and NAT10-knockdown GC cells (MKN45 and AGS) were generated (Figure 4C, S3C, S3D, S3E, and S3F). Moreover, we constructed NAT10-knockout AGS cells through clustered regularly interspaced short palindromic repeats (CRISPR)/Cas9 gene editing (Figure 4D, 4E, and S3G). As expected, the ac4C level was dramatically increased in NAT10-WT-overexpressing cells but not in catalytically inactive NAT10 mutant-overexpressing cells and was reduced in NAT10-knockout and NAT10-knockdown cells (Figure 4C, 4E, and S3H). To further elucidate the function of NAT10 in GC cells, we performed RNA sequencing (RNA-seq) analysis of GC cells with NAT10 overexpression or knockout. Notably, the glycolytic pathway was enriched, according to Kyoto Encyclopedia of Genes and Genomes (KEGG) enrichment analysis and gene set enrichment analysis (GSEA) (Figure 4F and S3I). Subsequently, we confirmed that overexpression of NAT10-WT but not either of the other two NAT10 mutants, significantly increased glucose uptake and lactate production, suggesting that the function of NAT10 in promoting glycolytic metabolism in GC cells depends on its enzymatic activity (Figure 4G and S3J). In contrast, knockout or knockdown of NAT10 markedly reduced glucose uptake and lactate production (Figure 4H and 4I). The extracellular acidification rate (ECAR) kinetic profiles further demonstrated that glycolytic activity was significantly increased in NAT10-overexpressing BGC823 cells but decreased in NAT10-knockout AGS cells (Figure 4J and 4K). To further examine the effects of NAT10 expression on glycolysis, 18F-FDG PET/CT was used to measure glucose uptake *in vivo*. The mouse PET/CT imaging results showed that overexpression of NAT10 dramatically increased glucose uptake but knockdown of NAT10 significantly inhibited glucose uptake in xenograft tumors (Figure 4L and 4M). Thus, our data indicate that NAT10-mediated promotion of glycolytic metabolism in GC cells is dependent on its ac4C catalytic activity.

### NAT10-mediated promotion of gastric tumorigenesis depends on the activation of glycolysis

**Figure 5:**
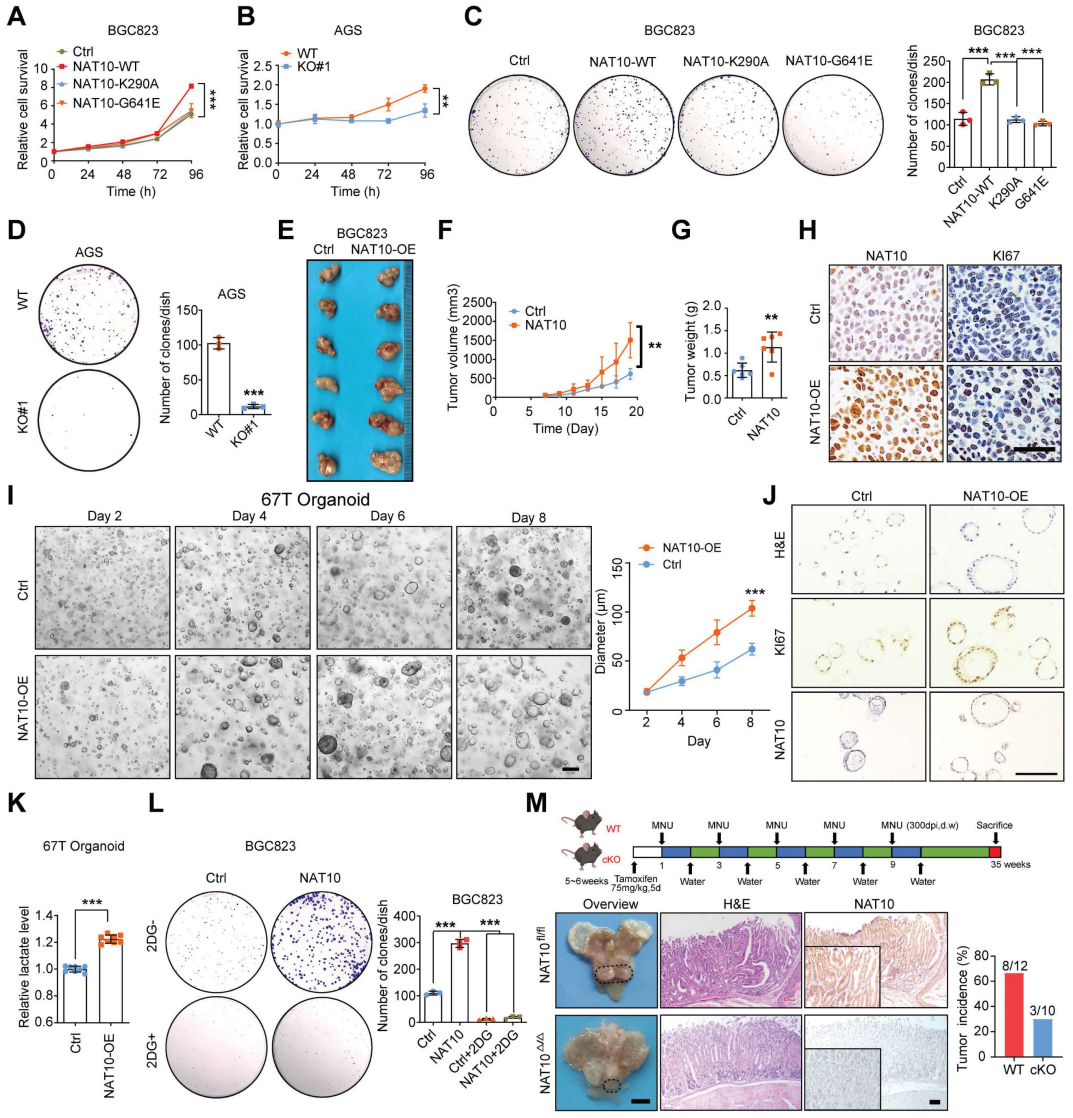
** NAT10 promotes gastric tumorigenesis in a manner dependent on the activation of glycolysis. (A)** A CCK8 assay was used to evaluate the proliferation of BGC823 cells overexpressing wild-type or catalytically dead mutant NAT10. **(B)** A CCK8 assay was used to evaluate cell proliferation in AGS WT or NAT10 KO cells. **(C)** A colony formation assay was performed in BGC823 cells with wild-type or catalytically dead mutant NAT10 overexpression (left panel). Quantitative analysis of the colony formation assay results (right panel). **(D)** A colony formation assay was performed in WT and NAT10 KO AGS cells (left panel). Quantitative analysis of the colony formation assay results (right panel). **(E)** Overexpression of NAT10 effectively promoted subcutaneous gastric tumor growth in nude mice (n = 6). **(F)** The tumor volume was monitored every other day, and tumor growth curves were generated.** (G)** The tumors were extracted and weighed after 20 days. **(H)** Sections of tumors from the NAT10-overexpressing and control groups were stained with anti-Ki-67 and anti-NAT10 antibodies for IHC analysis (scale bars = 50 μm).** (I)** Representative images of GC organoids transduced with the NAT10 overexpression or control lentiviral vector at the indicated time (scale bars = 100 μm, left panel) and quantification of organoid diameters (right panel). **(J)** Sections of organoids formed from cells transduced with the NAT10 overexpression or control lentiviral vector were subjected to H&E staining or stained with anti-Ki-67 and anti-NAT10 antibodies for IHC analysis (scale bars = 50 μm).** (K)** Lactate production in 67T organoids formed from control and NAT10 overexpression cells was measured. **(L)** A colony formation assay was performed with BGC823 control or NAT10-overexpressing cells subsequently treated with 5 mM 2-DG (2-deoxyglucose) (left panel); the colony formation assay results were quantitatively analysed (right panel). **(M)** Graphical illustration of MNU-induced GC tumors in mice (upper panel); Representative macroscopic images (scale bars = 0.5 cm), H&E staining images (scale bars = 100 μm), and NAT10 IHC staining images (scale bars = 100 μm) of stomachs from NAT10 WT and cKO mice (bottom panel). H&E, haematoxylin and eosin; IHC, immunohistochemical; GC, gastric cancer; WT, wild type; KO, knockout. The statistical data in this figure are presented as the mean value ± SD of three independent experiments. Statistical significance was determined by a two-tailed t test. * P < 0.05; ** P < 0.01; *** P < 0.001.

Subsequently, we evaluated the roles of NAT10 in GC* in vitro* and *in vivo*. First, we analysed the gene expression data from the GEPIA database (http://gepia.cancer-pku.cn/), which revealed that NAT10 expression was significantly correlated with the expression of KI-67 and PCNA (proliferating cell nuclear antigen), which are markers of cell proliferation (Figure S4A). As expected, in gain-of-function assays, overexpression of NAT10-WT but not either of the NAT10 mutants increased cell proliferation and colony formation *in vitro* (Figure 5A, 5C, S4B, and S4E). In contrast, knockout or knockdown of NAT10 obviously suppressed cell proliferation and colony formation (Figure 5B, 5D, S4C, S4D, and S4F). Furthermore, we verified the roles of NAT10 in GC cells *in vivo*, and the results showed that overexpression of NAT10 in GC cells significantly promoted tumor growth, as indicated by the larger size and greater weight of these tumors compared with those in the control group (Figure 5E, 5F, and 5G). In addition, the IHC results showed increased expression of NAT10 and KI-67 in the tumor tissues of the NAT10-overexpressing group (Figure 5H). Conversely, NAT10 knockdown in MKN45 cells produced the opposite effects *in vivo* (Figure S4G, S4H, S4I, and S4J). Furthermore, GC organoids were generated from samples from six different GC patients, and the NAT10 protein level was examined via an IHC assay (Figure S4K). Then, organoids with low NAT10 expression were infected with NAT10-overexpressing lentivirus or the corresponding control lentivirus. As expected, the ac4C abundance was dramatically increased in the NAT10-overexpressing organoids (Figure S4L). Moreover, overexpression of NAT10 significantly promoted the growth of tumor organoids, the diameter of the NAT10-overexpressing organoids was significantly increased compared with that of their counterparts (Figure 5I), and the IHC results showed increased expression of NAT10 and KI-67 in the NAT10-overexpressing organoids (Figure 5J). Furthermore, we confirmed that lactate production was significantly increased in the NAT10-overexpressing organoids than in the control organoids (Figure 5K). In addition, 2-DG, an inhibitor of glycolysis, significantly blocked NAT10-induced cell proliferation (Figure S4M) and colony formation (Figure 5L). To further evaluate whether glucose deficiency or autophagy activation inhibits GC progression by downregulating NAT10, we further constructed a subcutaneous xenograft model using GC cells overexpressing NAT10 and corresponding control cells, and treated these mice with 2-DG or rapamycin. The *in vivo* results showed that both 2-DG and rapamycin treatments significantly suppressed tumor growth, but overexpression of NAT10 could weaken the anti-tumor effects caused by 2-DG and rapamycin, as indicated by the decreases in tumor size and weight (Figure S4N, S4O, and S4P). In addition, the IHC results showed that both 2-DG and rapamycin treatment groups decreased expression of KI-67 and NAT10 in both the NAT10 overexpression group and control cell group the control group, while it was correspondingly increased in the NAT10 overexpression group compared to the control group (Figure S4Q). These data strongly suggest that NAT10 may play an oncogenic role and promote GC progression by regulating glucose metabolism.

To investigate the role of NAT10 in gastric carcinogenesis, we further generated NAT10-cKO mice using the Cre/LoxP recombinase system. ANXA10 has been reported to be specifically expressed in mouse gastric mucosal epithelial cells, and Anxa10-2A-CreERT2 has been used for specific knockout of target genes in the mouse gastric epithelium 43. Then, NAT10-cKO mice were generated and confirmed (Figure S4R). Tamoxifen specifically induced the emission of red fluorescence in gastric mucosal epithelial cells but not in other organs in Anxa10-2A-CreERT2 mice crossed with Rosa26-CAG-LSL-Cas9-tdTomato mice (Figure S4S). To determine the effect of NAT10 on GC development, MNU was applied to induce gastric tumorigenesis in WT mice and NAT10 cKO mice as described in a previous study 42. We found that specific knockout of NAT10 in mouse gastric mucosal epithelial cells significantly reduced the occurrence and development of GC compared to that in WT mice with gastric tumorigenesis induced by MNU (Figure 5M). These results indicate that NAT10 in gastric epithelial cells is essential for the development of GC in mice.

### NAT10-mediated ac4c modification of HK2 mRNA maintains its stability

**Figure 6:**
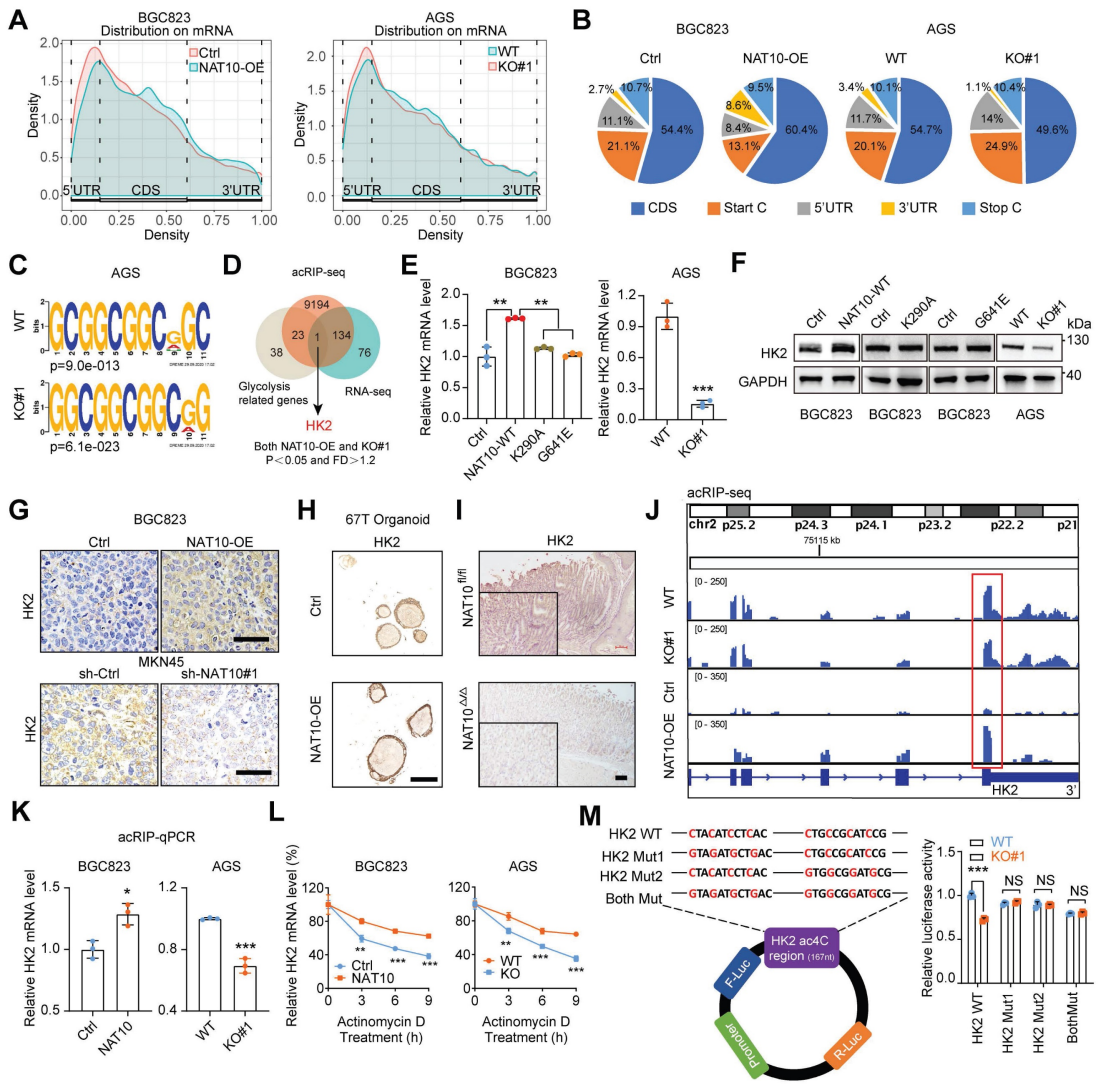
** NAT10-mediated ac4C modification of HK2 mRNA maintains its stability. (A)** The distribution of ac4C-containing peaks across mRNAs in NAT10-overexpressing (left panel) and NAT10 knockout (right panel) GC cells is displayed in a metagene plot. **(B)** Representative pie chart of the peak distribution showing the proportions of total ac4C peaks in the indicated regions, including the 5′-untranslated region (5′-UTR), coding sequence (CDS), 3′- untranslated region (3′-UTR), start codon, and stop codon. **(C)** Consensus motif in AGS WT and NAT10-knockout cells identified by HOMER. **(D)** Venn diagram showing HK2 as the selected candidate target gene of NAT10.** (E)** The mRNA level of HK2 in NAT10-overexpressing and NAT10-KO GC cells was measured by qRT-PCR. **(F)** The protein level of HK2 in NAT10-overexpressing and NAT10-KO GC cells was measured by Western blotting. **(G)** Sections of xenograft tumors derived from NAT10-overexpressing (upper panel) and NAT10-knockdown (bottom panel) GC cells subcutaneously injected into nude mice were stained with an anti-HK2 antibody for IHC analysis (scale bars = 50 μm). **(H)** Sections of organoids formed from cells transduced with the NAT10 overexpression or control lentiviral vector were subjected to H&E staining or IHC staining with an anti-HK2 antibody (scale bars = 50 μm). **(I)** Sections of stomachs from NAT10 WT and cKO mice were stained with an anti-HK2 antibody for IHC analysis (scale bars = 100 μm).** (J)** Integrative Genomics Viewer (IGV) tracks revealing the ac4Cseq read distribution on HK2 mRNA in NAT10-overexpressing and NAT10-knockout GC cells. **(K)** acRIP-qPCR analysis was employed to demonstrate the presence of NAT10-mediated HK2 ac4C modifications. ac4C modification of HK2 was increased upon overexpression of NAT10, while it was decreased upon knockout of NAT10.** (L)** The level of HK2 expression in NAT10-overexpressing, NAT10-knockout and the corresponding control GC cells treated with actinomycin D (2 μg/mL) at the indicated time points was measured by qRT-PCR. **(M)** Schematic representation of the construction of the luciferase reporter vectors containing the HK2 WT, Mut1, Mut2, or Both Mut sequences (left panel). The relative luciferase activities of the HK2 WT, Mut1, Mut2, and Both Mut luciferase reporters in AGS cells with NAT10 knockout and the corresponding control cells were measured (right panel). H&E, haematoxylin and eosin; IHC, immunohistochemical; GC, gastric cancer; WT, wild type; KO, knockout. The statistical data in this figure are presented as the mean values ± SDs of three independent experiments. Statistical significance was determined by a two-tailed t test. * P < 0.05; ** P < 0.01; *** P < 0.001.

To identify the molecular mechanism by which NAT10 promotes glycolysis and tumorigenesis in GC, we first conducted ac4C-modified RNA immunoprecipitation sequencing (acRIP-seq) in NAT10-overexpressing cells, NAT10 knockout cells and the corresponding control cells. The acRIP-seq results revealed that the ac4C peaks of 9352 transcripts exhibited significantly different abundances (fold change > 1.2, P < 0.05). The ac4C peaks were distributed mainly within the coding sequence (CDS), 3′ untranslated region (UTR), and 5′ UTR (Figure 6A and 6B). Consistent with the findings of previous studies, sequential analysis of the ac4C peaks revealed that typical '**C**XX**C**XX**C**XX**C**XX' motifs were highly enriched within ac4C-modified sites (Figure 6C). Considering that NAT10 regulates glucose metabolism, 66 glycolysis-related genes identified via the GSEA website (https://www.gsea-msigdb.org/gsea/msigdb/index.jsp) were evaluated as potential target genes. Intriguingly, HK2 was the only gene overlapping among the RNA-seq, acRIP-seq, and glycolysis-related genes (Figure 6D). Next, we validated the expression of HK2 in GC cells with NAT10 overexpression or knockdown. The mRNA and protein levels of HK2 were consistently regulated by NAT10 in all four GC cell lines (Figure 6E, 6F, S5A, and S5B). However, we found that the NAT10 mutants could not regulate HK2 expression at either the transcriptional or translational level (Figure 6E and 6F). Moreover, the IHC results confirmed that HK2 was consistently regulated by NAT10 in tumor xenograft tissues, organoids, and tissues of MNU-induced GC (Figure 6G, 6H, 6I, and S5C).

The ac4C abundance in HK2 mRNA was significantly decreased upon knockout of NAT10 but was obviously increased upon NAT10 overexpression, especially at the intersection between the CDS and 3'UTR (Figure 6J), indicating that ac4C modification might affect the stability of HK2 mRNA. The acRIP-qPCR results also showed that HK2 mRNA was significantly enriched by the ac4C-specific antibody upon NAT10 overexpression, while this enrichment was reduced upon NAT10 knockout (Figure 6K). Furthermore, the HK2 mRNA level was shown to be highly stable upon NAT10 overexpression, while instability was observed upon knockout of NAT10 in GC cells treated with actinomycin D, an inhibitor of transcription, for the indicated times (Figure 6L and S5D). To further verify that ac4C modification of HK2 mRNA occurred at the '**C**XX**C**XX**C**XX**C**XX' motif at the intersection between the CDS and 3'UTR, we replaced cytosine (C) bases with guanine (G) bases in two different motifs and inserted the WT and mutated sequences into luciferase reporter plasmids; the results of the subsequent luciferase reporter assay demonstrated that knockout of NAT10 caused a decrease in luciferase activity in the HK2-WT group but not in the HK2-Mut group (Figure 6M). Taken together, our findings indicate that NAT10-mediated ac4C modification maintains HK2 mRNA stability and increases its expression.

### NAT10 enhances glycolysis and accelerates tumor progression in GC by upregulating HK2 expression

**Figure 7:**
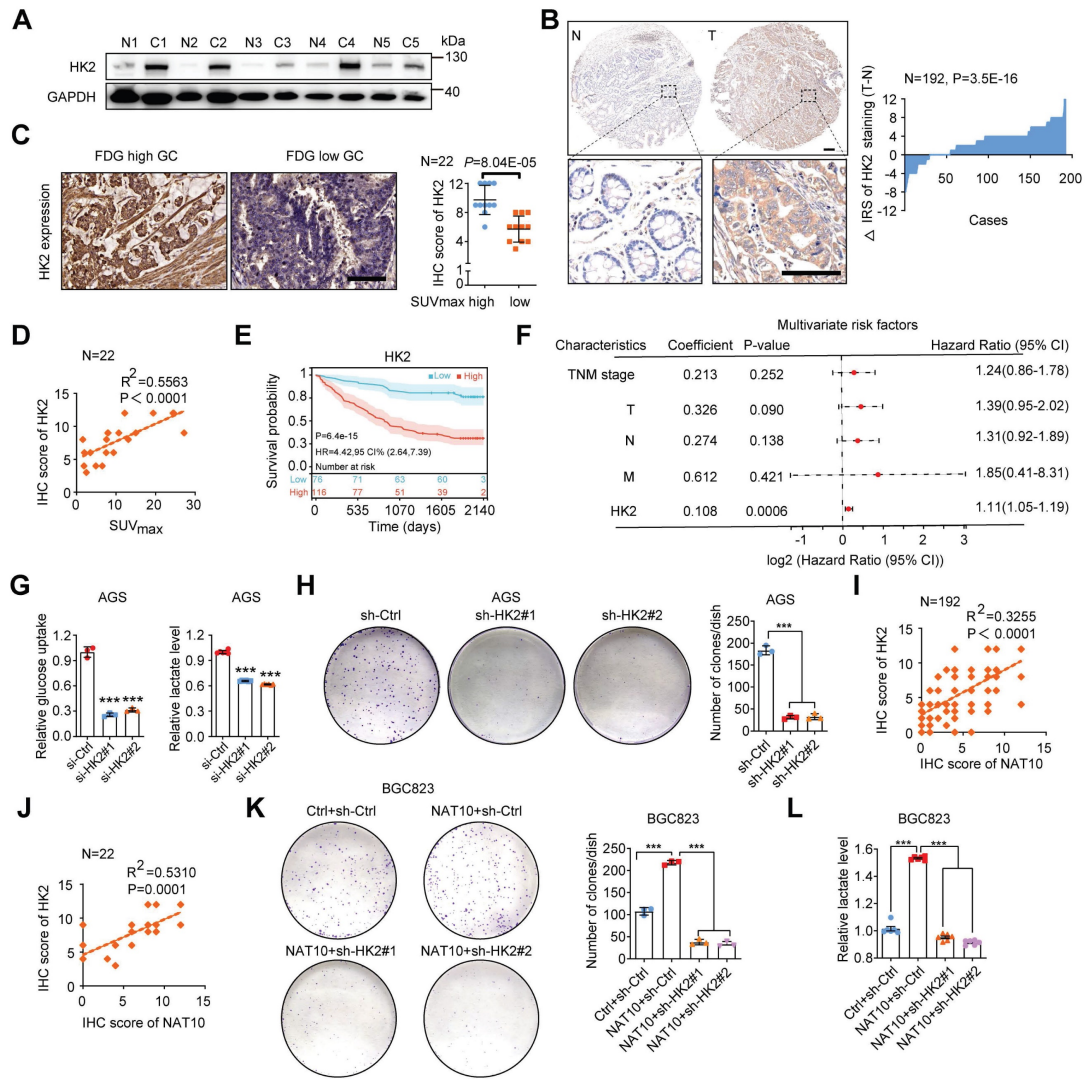
** NAT10 accelerates malignant progression of GC by upregulating HK2. (A)** The HK2 protein level was measured in GC tissues and paired normal gastric mucosal tissues by Western blotting (n = 5). **(B)** Representative IHC images of the tissue microarray analysed with the anti-HK2 antibody (scale bars = 200 or 100 μm) are shown (left panel). The distribution of the difference in the HK2 immunoreactivity score (IRS) (△IRS = IRST - IRSN) is shown. The IRS for HK2 staining was available for 192 pairs of tissues. **(C)** Representative images of HK2 IHC staining in tissues from GC patients in the SUVmax-high and SUVmax-low groups (left panel). Scale bars = 50 μm; n = 22; statistical analysis of the difference in HK2 expression between the SUVmax-high group and the SUVmax-low group (right panel). **(D)** Correlation analysis between the SUVmax and HK2 protein expression level based on the IHC score in GC tissue microarrays with associated PET/CT data. R, Pearson correlation coefficient; n = 22. **(E)** Kaplan-Meier analysis of OS in GC patients stratified by HK2 expression (n = 192, P = 6.4e-15; log-rank test). **(F)** Multivariate analyses were performed for the GC cohort. **(G)** Glucose uptake (left panel) and lactate production (right panel) in HK2-knockdown AGS cells were measured. **(H)** A colony formation assay was performed in stable HK2-knockdown AGS cells (left panel). Quantitative analysis of the colony formation assay results (right panel). **(I)** NAT10 expression was positively correlated with HK2 protein expression in GC tissues (linear regression) according to analysis of the IHC score from the TMA data (n = 192). **(J)** Correlation analysis of NAT10 and HK2 protein expression based on the IHC score in GC tissue microarrays with associated PET/CT data (n = 22).** (K)** The colony formation ability was evaluated in NAT10-overexpressing BGC823 cells with or without stable HK2 knockdown or the corresponding controls. Representative images (left panel) and quantitative results (right panel) are shown. **(L)** Lactate production was measured in NAT10-overexpressing BGC823 cells with or without stable HK2 knockdown or the corresponding controls. CI, confidence interval; GC, gastric cancer; HR, hazard ratio; IHC, immunohistochemical; OS, overall survival; TNM, tumor, node, metastasis. The statistical data in this figure are presented as the mean value ± SD of three independent experiments. Statistical significance was determined by a two-tailed t test. * P < 0.05; ** P < 0.01; *** P < 0.001.

HK2 is one of the key rate-limiting enzymes in the glycolytic pathway and is consistently overexpressed in various cancers, including GC 6, 44. However, the relationship between HK2 expression and the prognosis of GC patients remains unclear. Here, we first found that HK2 mRNA expression was significantly upregulated in GC via analysis of TCGA data (Figure S6A). Furthermore, analysis of fresh GC tissues and TMAs confirmed that the HK2 protein level was significantly increased in GC tissues (Figure 7A and 7B). As expected, we also found a significant correlation between FDG uptake and HK2 expression in 22 GC patients (Figure 7C and 7D). Analysis of data from our GC cohort also showed that GC patients with increased HK2 expression had worse OS (n = 192, P = 6.4E-15; log-rank test; Figure 7E). Furthermore, in patients with GC, univariate Cox regression analysis revealed substantial associations between survival and TNM stage; individual T, N, and M classifications; and HK2 expression (Figure S6B). In addition, multivariate Cox regression analysis indicated that HK2 expression was an independent predictor of prognosis in patients with GC (Figure 7F). As expected, knockdown of HK2 by its specific siRNAs in AGS cells markedly reduced glucose uptake and lactate production, while stable overexpression of HK2 in BGC823 cells significantly increased glucose uptake and lactate production (Figure 7G, S6C, S6E, and S6F). To further confirm the function of HK2, we constructed a stable cell line with low expression of HK2 using its specific shRNAs lentivirus (Figure S6D). Consistent with these findings, HK2 knockdown obviously suppressed cell proliferation, while HK2 overexpression increased cell proliferation, as determined via a colony formation assay (Figure 7H and S6G). Furthermore, we found that the mRNA level of NAT10 was significantly correlated with that of HK2 via analysis of TCGA data (Figure S6H). A positive correlation between the protein levels of NAT10 and HK2 was also validated using a TMA (R^2^ = 0.3255, P < 0.0001; Figure 7I). NAT10 expression was positively correlated with HK2 expression in 22 patients with GC, as determined by 18F-FDG PET/CT (R^2^ = 0.5310, P = 0.0001; Figure 7J). Furthermore, HK2 expression was stably knocked down in NAT10-overexpressing BGC823 cells using its specific shRNAs lentivirus, and HK2 knockdown markedly suppressed NAT10-induced GC cell colony formation and lactate production (Figure 7K, 7L, and S6I). Consistently, HK2 stably overexpression rescued the colony forming ability of NAT10 knockdown GC cells (Figure S6J and S6K). Thus, our data suggest that NAT10 promotes malignant progression of GC through HK2-mediated glycolysis.

### The clinical significance of the NAT10/HK2 axis in GC

**Figure 8:**
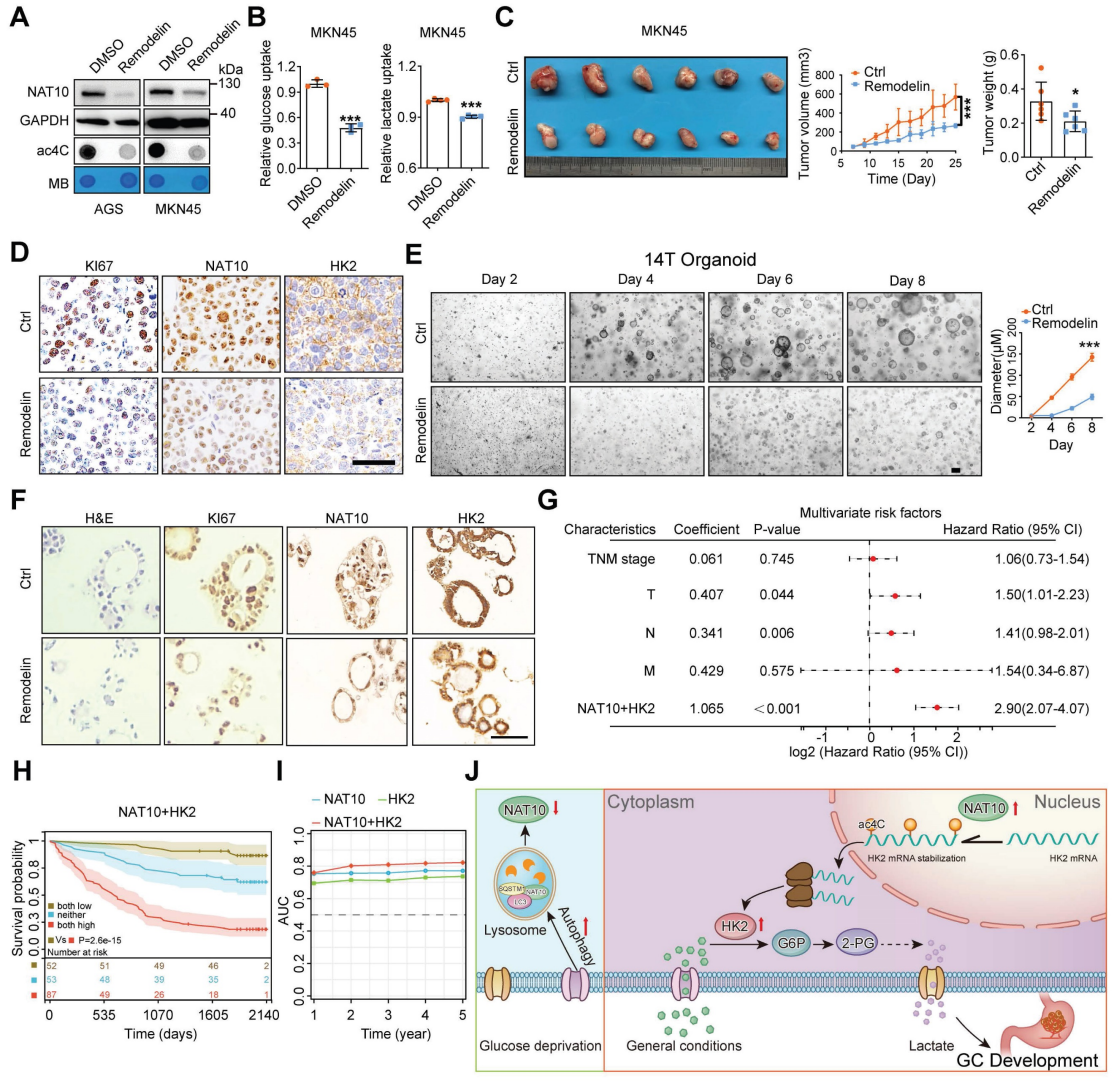
** Targeting the NAT10-HK2 axis in GC cells has clinical value. (A)** Different GC cell lines were treated with 10 μM remodelin for 24 h. Then, the protein level of NAT10 was measured by Western blot analysis (upper panel). mRNA isolated from GC cells were subjected to dot blot analysis with an anti-ac4C antibody (bottom panel). MB staining served as a loading control. **(B)** Glucose uptake (left panel) and lactate production (right panel) were measured in MKN45 cells after treatment with 10 μM Remodelin for 24 h.** (C)** Remodelin inhibited subcutaneous tumor growth in nude mice (n = 6, left panel). The tumor volume was monitored every other day, and tumor growth curves were generated (middle panel). The tumors were extracted and weighed after 25 days (right panel). **(D)** Sections of tumors were stained with anti-Ki-67, anti-HK2, and anti-NAT10 antibodies for IHC analysis (scale bars = 50 μm). **(E)** Representative images of GC organoids treated with 10 μM Remodelin for the indicated durations (scale bars = 100 μm, left panel) and quantification of organoid diameters (right panel). **(F)** Sections of organoids treated with Remodelin were subjected to H&E staining or stained with anti-Ki-67, anti-HK2, and anti-NAT10 antibodies for IHC analysis (scale bars = 50 μm). **(G)** The combination of NAT10 and HK2 was evaluated as a new two-gene risk signature, and multivariate analyses were performed for the GC cohort. **(H)** GC patients were divided into three subgroups according to the median expression level of each protein: high expression of both NAT10 and HK2, low expression of both NAT10 and HK2, and other expression patterns (NAT10 high and HK2 low OR NAT10 low and HK2 high). Kaplan-Meier analysis of survival in the three subgroups of GC patients. **(I)** Time-dependent receiver operating characteristic (ROC) curve analysis of the NAT10 risk score, the HK2 risk score, and the combined NAT10/HK2 score in the GC cohort. AUC, area under the curve; CI, confidence interval; GC, gastric cancer; HR, hazard ratio; IHC, immunohistochemical; OS, overall survival; TNM, tumor, node, metastasis. **(J)** Graphical illustration of the mechanism by which NAT10 modulates glycolysis, promoting GC growth. The statistical data in this figure are presented as the mean value ± SD of three independent experiments. Statistical significance was determined by a two-tailed t test. * P < 0.05; ** P < 0.01; *** P < 0.001.

To investigate the clinical significance of NAT10, we subsequently evaluated the anticancer activity of the NAT10-specific inhibitor remodelin 45. Remodelin exhibited some degree of cytotoxicity in GC cell lines at high concentrations (Figure S7A). However, treatment with remodelin at a nontoxic concentration dramatically decreased the NAT10 protein level and the ac4C level but did not affect the mRNA level of NAT10 in GC cells (Figure 8A and S7B). Furthermore, treatment with remodelin at a nontoxic concentration markedly reduced glucose uptake and lactate production (Figure 8B). Next, we validated the expression of HK2 after remodelin treatment and found that remodelin inhibited the expression of HK2 (Figure S7C). As expected, treatment with remodelin at a nontoxic concentration obviously suppressed cell proliferation and colony formation *in vitro* (Figure S7D). Meanwhile, we found that Remodelin could also inhibit the proliferation of GC cells even in low glucose conditions (Figure S7E). Interestingly enough, our results also showed that Remodelin could increase sensitivity to chemotherapy drugs (cisplatin and 5-fluorouracil, 5-FU) in GC (Figure S7F). Furthermore, the *in vivo* results also showed that, compared with that in the vector control group, remodelin significantly suppressed tumor growth, as indicated by the decreases in tumor size and weight (Figure 8C). In addition, the IHC results showed decreased expression of NAT10 and KI-67 in the tumors of the remodelin group compared with the control group (Figure 8D). Furthermore, we evaluated tissue toxicity using haematoxylin and eosin (H&E) staining, which revealed no significant toxicity to major organs, including the heart, liver, spleen, lungs, and kidneys (Figure S7G).

Additionally, we confirmed the inhibitory effect of remodelin in GC organoids and showed that remodelin decreased both the NAT10 protein level and the ac4C level in GC organoids with high NAT10 expression (Figure S4K and S7H). Moreover, remodelin significantly inhibited the growth of GC organoids compared with that in the control group (Figure 8E). IHC also revealed decreased expression of NAT10, HK2, and KI-67 in the organoids in the remodelin group compared with those in the control group (Figure 8F).

Next, we analysed the prognostic value of high NAT10 expression combined with high HK2 expression, and univariate/multivariate Cox regression analyses showed that the risk score of the combination (univariate: HR = 3.63, P < 0.001; multivariate: HR = 2.90, P < 0.001) was higher than that of either NAT10 alone (HR = 1.22, P < 0.001; HR = 1.16, P < 0.001, respectively) or HK2 alone (HR = 1.17, P < 0.001; HR = 1.11, P < 0.001, respectively) (Figure 8G and S7I). Moreover, GC patients were divided into three subgroups according to the median expression level of each protein: high expression of both NAT10 and HK2, low expression of both NAT10 and HK2, and other expression patterns. The Kaplan-Meier curves demonstrated that GC patients with high levels of both NAT10 and HK2 had worse outcomes than the other patients (Figure 8H). In addition, to further evaluate the predictive ability of NAT10 and HK2 expression, we conducted time-dependent receiver operating characteristic curve analysis, which indicated that the combination of NAT10 and HK2 had better predictive potential than either alone in the GC cohort (Figure 8I).

## Discussion

With the advancements in analytical chemistry and high-throughput sequencing-related technologies, new technologies and methods for exploring RNA modifications are constantly being developed. Many different RNA modifications are reportedly involved in the occurrence and development of various diseases, including cancer 14, 46, 47. RNA modifications, including m1A, m5C, m6A, m7G, and ac4C, mainly affect RNA metabolism 14, 46. In the past few years, the biological function of m6A, the most common RNA modification, has been widely and deeply studied, while the study of other RNA modifications is in its infancy 18, 48, 49. Recent studies have confirmed the presence of ac4C modifications on human and yeast mRNAs, with beneficial effects on the stability and translation efficiency of the modified mRNAs 50. In the present study, we first identified crosstalk between ac4C modification and glucose metabolism in gastric tumorigenesis. On the one hand, the autophagy pathway is activated to promote the formation of the NAT10/SQSTM1/LC3 complex, leading to the degradation of NAT10 through the lysosomal pathway upon glucose deprivation. On the other hand, upregulation of NAT10 significantly increased ac4C modification in GC. Subsequently, NAT10 promoted glucose metabolism via ac4C modification of HK2 mRNA to drive gastric tumorigenesis (Figure 8J).

Recent reports have consistently shown that ac4C modification plays important and diverse biological roles in various physiological and pathological processes, including cancer 28, 29, 31. Previous studies have showed that NAT10 controls cell fates via connecting mRNA ac4C modification to chromatin signaling 51, and the physiological functions of ac4C modifications in male spermatogenesis 52. It has been shown that NAT10 can mediate ac4C modification of COL5A1 mRNA and enhance its stability, promoting metastasis and EMT in GC 53. Another study indicated that *Helicobacter pylori*-induced NAT10 can stabilize MDM2 mRNA via acetylation to facilitate GC progression 31.

Recently, a study revealed the relationship between mRNA ac4C modification and hypoxia, suggesting that the NAT10/SEPT9/HIF-1a loop is involved in GC progression 29. Here, we first revealed that glucose status could control NAT10-mediated ac4C modification in GC. It is the first to propose that in tumors, NAT10 may be degraded via the autophagy-lysosome pathway following glucose stress, and we have also revealed that the upstream regulatory role of NAT10 from the perspective of post-translational modification. Moreover, our results revealed that the ac4C modification and NAT10 expression levels are increased in GC and that NAT10 expression is associated with poor prognosis in patients with GC; furthermore, the inclusion of NAT10 expression can increase the predictive ability of clinical risk scores, suggesting that NAT10 may be a diagnostic biomarker for GC. Additionally, there was a notable correlation between NAT10 expression and 18F-labeled glucose uptake. We further confirmed that high NAT10 expression promotes glucose uptake and metabolism *in vivo*. This is the first study to utilize clinical PET/CT data from GC patients and *in vivo* PET/CT detection in mice to analyze the correlation between NAT10 expression and glucose metabolism, providing an excellent biomarker and molecular target for the diagnosis and treatment of clinical GC patients. Subsequently, *in vitro* and *in vivo* studies showed that the promotion of GC tumor growth by NAT10 was dependent on the activation of glycolysis. Furthermore, we investigated the role of NAT10 in MNU-induced gastric carcinogenesis using NAT10-cKO mice, and the results showed that specific knockout of NAT10 in mouse gastric mucosal epithelial cells significantly reduced the occurrence and development of GC. Thus, NAT10 in gastric epithelial cells is essential for the development of GC and could be a potential predictive biomarker and therapeutic target for GC. This study is the first to report on tumorigenesis using a conditional knockout mouse model for NAT10, providing direct evidence from an *in vivo* model for the crucial role of NAT10 in GC development.

Using RNA-seq and acRIP-seq, we found promising results indicating that HK2 is the key downstream target of NAT10 in GC. HK2 is a key metabolic enzyme that catalyses the first step of the glycolytic pathway, phosphorylating glucose to generate glucose 6-phosphate, and is localized mainly on the outer membrane of mitochondria 11. HK2 is highly expressed in tumors and plays an important role in promoting the Warburg effect during cancer-related processes, including cancer growth, apoptosis, angiogenesis and metastasis 54-57. Recently, HK2 was shown to perform a new function that is independent of its classical metabolic function: HK2 can act as a protein kinase to mediate the phosphorylation of IκBα and activate the NF-κB pathway, promoting the expression of PD-L1 and tumor immune escape 58. In our study, we found that NAT10 can maintain HK2 mRNA stability and increase HK2 mRNA expression via ac4C modification of its CDS and 3'UTR in GC cells. Our data also suggested that NAT10 promotes malignant progression of GC through HK2-mediated glycolysis. Furthermore, the expression of NAT10 was positively correlated with the expression of HK2 in GC tissues, indicating the clinical significance of the NAT10/HK2 axis in promoting glycolysis in GC. Our data reveal that the regulatory mechanism is entirely different from previous study, and our research starts from the regulation of NAT10 by glucose homeostasis to the direct regulation of the first rate-limiting enzyme gene HK2 in the glycolysis pathway through NAT10-mediated mRNA ac4C modification. This consistent approach demonstrates the importance of NAT10 in regulating gastric cancer malignancy progression through glucose metabolic homeostasis.

## Conclusion

In summary, our findings reveal that glucose homeostasis controls NAT10-mediated ac4C modification in GC. The expression of the NAT10/HK2 axis components is significantly increased in GC and is correlated with poor prognosis in patients with GC. The NAT10/HK2 axis promotes gastric tumorigenesis via increasing glycolysis. Therefore, the NAT10/HK2 axis could be a potential prognostic predictor and therapeutic target for GC. However, further research using multiple models is needed to fully elucidate the potential mechanisms and verify whether there is heterogeneity or similarity in different subtypes of GC. At the same time, future research should explore the combination of NAT10/HK2 inhibitors with existing therapies (immunotherapy, chemotherapy, targeted therapy, etc.) to improve treatment efficacy and develop biomarkers for personalized medical methods of GC.

## Materials and Methods

### Patients and specimens

The cohort of patients with GC treated with radical gastrectomy without adjuvant radiotherapy or chemotherapy was enrolled from Nanjing Drum Tower Hospital, the Affiliated Hospital of Nanjing University Medical School (Nanjing, Jiangsu, China). The GC cohort contained 192 patients who underwent radical gastrectomy between January 14, 2014, and January 4, 2016. The median survival time was 56 months. Paired gastric tumor and normal gastric mucosal tissue specimens were embedded in paraffin to construct a tissue microarray (TMA), and clinicopathological features of the patients, including age, sex, and TNM stage (American Joint Committee on Cancer (AJCC) classification), were recorded. Additionally, fresh-frozen pathologically confirmed gastric tumor and normal gastric mucosal tissue specimens from recent patients at Nanjing Drum Tower Hospital were included. These tissues were obtained for qRT‒PCR analysis, Western blot analysis, and GC organoid culture after written informed consent was provided. This study was approved by the Institutional Review Board (IRB) of Nanjing Drum Tower Hospital, and all participants provided written informed consent prior to their participation in this study.

### Cell lines and culture

The AGS and NCI-N87 GC cell lines were purchased from the American Type Culture Collection (MD, USA), and the SGC7901, BGC823, HGC27, and MGC803 GC cell lines were obtained from the Type Culture Collection of the Chinese Academy of Sciences (TCCCAS, Shanghai, China). MKN-45 cells were obtained from the cell bank of the RIKEN BioResource Center (Tsukuba, Japan). SGC7901, NCI-N87, BGC823, and MKN45 cells were cultured in RPMI-1640 medium (Invitrogen Life Technologies, CA, USA), MGC803 cells were cultured in DMEM (Invitrogen Life Technologies, CA, USA), and AGS cells were cultured in F12K medium (Cellcook Biotech Co., Ltd, Guangzhou, China). All culture media were supplemented with 10% foetal bovine serum (FBS; Wisent, Montreal, Canada), 100 μg/ml streptomycin, and 100 U/ml penicillin (New Cell & Molecular Biotech, Suzhou, China), and all cells were cultured in an incubator with 5% CO^2^ at 37 °C. The cells were stored at -80 °C using CELLSAVING (New Cell & Molecular Biotech, Suzhou, China). All cells were tested negative for mycoplasma contamination and were authenticated based on STR fingerprinting before use. All the agents, inhibitors or agonists used in the studies described in this manuscript are listed in Supplementary Table 1.

### GC mouse xenograft model

Male BALB/c nude mice (5-6 weeks old) were purchased from Nanjing Biomedical Research Institute of Nanjing University (Nanjing, Jiangsu, China). The indicated GC cells (2×10^6^ BGC823; 3×10^6^ MKN45) were subcutaneously injected into the right axillae of the mice. Tumor volume was monitored every other day (volume = length × width^2^ × 1/2). 2DG (MCE, NJ, USA) and Rapamycin (MCE, NJ, USA) were injected intraperitoneally once a day when the tumor grows to 50-100mm^3^. At the indicated time, the mice were sacrificed, and the tumors were weighed and imaged. Tumor tissues were then fixed in 4% paraformaldehyde or frozen for further analyses. All animal experiments were performed in accordance with a protocol approved by the Institutional Animal Care Committee of Nanjing Drum Tower Hospital.

### Generation of NAT10 cKO mice and establishment of the spontaneous GC model

Nat10-flox mice (strain no. T007971) were purchased from GemPharmatech (Nanjing, China). According to the structure of the Nat10 gene, exon 4 to exon 5 of the Nat10-201 (ENSMUST00000028608.12) transcript is recommended for use as the knockout region. This region contains a 295 bp coding sequence. Deletion of this region results in disruption of protein function. In this project, we used CRISPR/Cas9 gene editing to modify the Nat10 gene sequence. Briefly, the CRISPR/Cas9 system and donor DNA were microinjected into fertilized eggs of C57BL/6JGpt mice. The fertilized eggs were transplanted to obtain positive F0 mice, which were confirmed by PCR and sequencing. Stable F1 mouse model was generated by mating positive F0 mice with C57BL/6JGpt mice. The floxed allele was knocked out in mice obtained by mating the F1 mice with mice expressing ANXA10 Cre (Anxa10-2A-CreERT2) recombinase, resulting in loss of function of the target gene in the gastric mucosal epithelial cells of the resulting mice. Genotyping of NAT10-cKO mice and WT mice was performed using PCR. The sequences of the primers used for PCR genotyping are listed in Supplementary Table 2.

The spontaneous GC model was established based on a published protocol 41, 42. Briefly, C57BL/6 mice were exposed to 240 ppm N-methyl-N-nitrosourea (MNU) in the drinking water for one full week every other week (total exposure time: five weeks). All mice were sacrificed after 35 weeks, and the stomachs were excised and washed with PBS. Subsequently, the stomachs were opened along the great curvature, flattened with fine needles, fixed, and imaged as whole-mount specimens before being embedded for histological examination.

### Organoid culture

The organoid culture method was described in our previous study 9. Briefly, approximately 1 cm^3^ of GC tissue from individual GC patients was minced, washed with 1× chelating buffer (5.6 mM Na2HPO4, 8.0 mM KH2PO4, 96.2 mM NaCl, 1.6 mM KCl, 43.4 mM sucrose, 54.9 mM D-sorbitol, and 0.5 mM DL-dithiothreitol (pH = 7)), and cut into 20-50 small pieces. The glands were compressed and then centrifuged for 5 min at 200 × g and 4 °C. Approximately 100 glands per 50 µl of basement matrix were seeded in one well of a 24-well plate warmed to 37 °C. Five hundred microlitres of medium containing growth factors (50 ng/ml EGF, 100 ng/ml noggin, 1 μg/ml R-spondin1, 50% Wnt-conditioned medium, 200 ng/ml FGF10, 1 nM gastrin, 2 µM TGF-beta inhibitor and 10 µM RHOKi) was carefully added to each well. After the organoids formed, we evaluated the NAT10 expression level in the organoids, and the suitable organoids were transfected with the NAT10 overexpression vector or the corresponding control lentiviral vector or were treated with the NAT10 inhibitor remodelin. Then, the organoids were seeded in 24-well plates and cultured for 8 days, after which images were acquired with a Leica DMi8 system at the indicated time. Then, the organoids were fixed and subjected to H&E staining, and the expression of NAT10, HK2, and Ki67 was evaluated using previously described methods 59, 60. The organoid culture procedure was first approved by the Institutional Review Board of Nanjing Drum Tower Hospital.

### SiRNA, shRNA, sgRNA, and plasmid transfection and lentiviral transduction

siRNAs targeting NAT10 or HK2 were designed and synthesized by RiboBio (Guangzhou, China). The sequences are listed in Supplementary Table 3. shRNAs targeting NAT10 or HK2 were designed based on the siRNA sequences and were subcloned and inserted into pLVX vectors (pLVX-puro), which were constructed by YouBio (Changsha, China). The NAT10-OE lentiviruses were produced by GeneChem Co., Ltd (Shanghai, China) using GV341 vectors (Ubi-MCS-3FLAG-SV40-puromycin). NAT10-OE lentiviruses expressing the NAT10 G641E or K290A mutant were produced based on the wild-type NAT10-OE construct by Corues Biotechnology (Nanjing, China). The HK2 lentiviral vector was constructed by YouBio (Changsha, China) using the PCDH-CMV-MCS-EF1-Hygro plasmid. siRNAs were transfected into cells with DharmaFECT4 (Dharmacon, Chicago, USA). All of the plasmids were transfected into cells with Lipofectamine 3000 (Invitrogen, Grand Island, NY, USA). Lentiviral shRNAs and overexpression plasmids and the corresponding control plasmids were transduced into GC cells. Eight to 12 hours later, the lentiviral culture medium was removed, and new culture medium was added. After 72 h, successfully transduced GC cells were selected with 1 μg/ml puromycin (Sigma, USA). To generate NAT10-knockout (NAT10-KO) AGS cells, the NAT10 sgRNA (Supplementary Table 3) was designed, cloned and inserted into a plasmid coexpressing the sgRNA and Cas9 (pCas-puro-U6-KO) by Corues Biotechnology (Nanjing, China), and NAT10-KO cells were generated as previously reported 22.

### Immunohistochemical (IHC) and TMA analyses

A standard protocol was used for IHC analysis, as described in a previous study 59, 60. Staining of NAT10 and HK2 in the tissues from the cohort was independently scored by two pathologists blinded to the clinical data by applying a semiquantitative immunoreactivity score (IRS), as reported previously 59, 60. Under these conditions, samples with an IRS of 0-4 and an IRS of 6-12 for NAT10 or HK2 were classified as having low and high expression, respectively, of the corresponding protein.

### Micro-PET/CT imaging of mice

The indicated GC cells (2×10^6^ BGC823; 3×10^6^ MKN45) were subcutaneously injected into the right axillae of BALB/c nude mice (5-6 weeks old). At the indicated time, tumor-bearing mice were deprived of food and water for 8-10 h before micro-PET/CT imaging. The mice were then injected with approximately 300 mCi of 18F-FDG via the tail vein, and 1 h later, scanning was performed with an Inveon micro-PET/CT system (Siemens Medical Solution). Isoflurane-anaesthetized mice were subjected to a 10-min micro-CT scan followed by a 10-min micro-PET scan. Inveon Research Workplace software was used to quantify the percentage of injected dose per gram (%ID/g) and the SUV. The SUVmax data were plotted and analysed.

### Protein mass spectrometry analysis

To identify the interacting proteins, BGC823 cells stably overexpressing Flag-NAT10 were cultured with 25 mM glucose medium or glucose-free medium containing leupeptin for 6 h. Then, the extracted proteins were separated via SDS‒PAGE and visualized via Coomassie blue staining. Then, the gel pieces were excised from the SDS-PAGE gels, destained for 20 min in 100 mM NH4HCO3 with 30% acetonitrile and washed with Milli‒Q water until the gels were destained. The gel plugs were then lyophilized in a vacuum centrifuge. Disulfide bonds in the in-gel proteins were reduced with dithiothreitol (10 mM DTT/100 mM [NH4]HCO3) for 30 min at 56 °C and then alkylated with iodoacetamide (200 mM IAA/100 mM [NH4]HCO3) in the dark at room temperature for 30 minutes. The gel pieces were briefly rinsed with 100 mM [NH4]HCO3 and ACN. The gel pieces were digested overnight with 12.5 ng/μl trypsin in 25 mM [NH4]HCO3. The peptides were extracted three times with 60% ACN/0.1% TFA. The extracts were pooled and dried completely by a vacuum centrifuge.

LC‒MS/MS: The peptides derived from each sample were desalted on C18 cartridges (Empore™ SPE Cartridges, Sigma), concentrated by vacuum centrifugation and reconstituted in 10 µl of 0.1% (v/v) formic acid. MS analysis was performed on a Q Exactive HF mass spectrometer that was coupled to an Easy nLC system (Thermo Scientific). Peptides were first loaded onto a trap column (100μm*20 mm, 5 μm, C18) with 0.1% formic acid and were then separated on an analytical column (75 μm*100 mm, 3 μm, C18) with a binary gradient of buffer A (0.1% formic acid) and buffer B (84% acetonitrile and 0.1% formic acid) at a flow rate of 300 nL/min over 60 min. The gradient was set as follows: 5%-8% buffer B from 0 min to 2 min, 8% to 23% buffer B from 2 min to 42 min, 23% to 40% buffer B from 42 min to 50 min, 40% to 100% buffer B from 50 min to 52 min, and 100% buffer B to 60 min. MS data were acquired using data-dependent top20 mode with dynamic selection of the most abundant precursor ions from the survey scan (350-1800 m/z) for HCD fragmentation. A lock mass of 445.120025 Da was used as an internal standard for mass calibration. The full MS scans were acquired at a resolution of 60,000 at m/z 200, and a resolution of 15,000 at m/z 200 was used to acquire the MS/MS scans. The maximum injection time was set to 50 ms for MS acquisition and 45 ms for MS/MS acquisition. The normalized collision energy was set to 27, and the isolation window was set to 1.5 Th. The dynamic exclusion duration was 30 s.

Database search: The MS data were analysed using MaxQuant software version 1.5.8.3. The MS data were searched against the UniProtKB Human database (157600 total entries, downloaded 07/2017). Trypsin was used as the digestion enzyme. The two maximal missed cleavage sites and a mass tolerance of 4.5 ppm for precursor ions and 20 ppm for fragment ions were defined as the parameters for the database search. For the database search, carbamidomethylation of cysteines was defined as the fixed modification, while acetylation of the protein N-terminus and lysines and oxidation of methionines were set as the variable modifications. The database search results were filtered and exported with a false discovery rate (FDR) of < 1% at the peptide and protein levels. LC‒MS/MS was completed by Shanghai Bioprofile (Shanghai, China).

### RNA sequencing (RNA-seq)

For RNA-seq, total RNA was first extracted from the indicated GC cells. The quality and quantity of the RNA were assessed by a NanoDropTM ND-1000 spectrophotometer. Denaturing agarose gel electrophoresis was used to assess RNA integrity. mRNA extraction was performed using an NEBNext Poly(A) mRNA Magnetic Isolation Module. RNA libraries were constructed using a KAPA Stranded RNA-Seq Library Prep Kit (Illumina). Libraries were sequenced using the Illumina HiSeq 4000 platform. RNA-seq was completed by GENE DENOV (Guangzhou, China).

### Ac4C-modified RNA immunoprecipitation sequencing (acRIP-seq)

For acRIP-seq, more than 150 µg of purified total RNA was obtained, and the integrity and quantity of each RNA sample were assessed via agarose gel electrophoresis and a NanoDropTM spectrophotometer. Intact mRNA was first isolated from total RNA samples using an Arraystar Seq-StarTM poly(A) mRNA Isolation Kit according to the manufacturer's protocol. The isolated mRNA was chemically fragmented into 100-nucleotide-long fragments by incubation in fragmentation buffer (10 mM Zn2+ and 10 mM Tris-HCl (pH 7.0)), and the size of the fragmented mRNA was confirmed via agarose gel electrophoresis. Then, ac4C-modified mRNAs were immunoprecipitated with an anti-ac4C antibody (an aliquot of the fragmented mRNAs was kept as input). The major downstream procedures included immunoprecipitation, washing, and elution. The eluted ac4C mRNA fragments were subsequently concentrated for RNA-seq library construction. RNA-seq libraries for the ac4C antibody-enriched mRNAs and input mRNAs were prepared using a KAPA Stranded mRNA-seq Kit (Illumina). The prepared libraries were diluted to a final concentration of 8 pM, and clusters were generated on an Illumina cBot system using a HiSeq 3000/4000 PE Cluster Kit (#PE-410-1001, Illumina) prior to sequencing on the Illumina HiSeq 4000 platform. For acRIP-seq data analysis, the raw reads were trimmed with Trimmomatic software and aligned to the Ensembl reference genome with HISAT2 software (v2.1.0). The differentially enriched regions (peaks) identified by acRIP-seq between the groups were analysed with exomePeak software. These differential peaks were annotated using the latest Ensembl database. Sequence motifs are one of the basic functional units of molecular evolution. The Multiple EM for Motif Elicitation (MEME) and Discriminative Regular Expression Motif Elicitation (DREME) algorithms were used to identify motifs in the ac4C peak sequences. acRIP-seq was completed by CloudSeq (Shanghai, China).

### Western blot analysis and coimmunoprecipitation (co-IP)

Western blot analysis: Total protein was extracted from tissues or cells with RIPA buffer containing protease inhibitors. The cell lysates were clarified by centrifugation at 12,000 rpm and 4 °C for 15 min. The protein concentrations were determined with a BCA Protein Assay Kit according to the manufacturer's instructions (Beyotime, Shanghai, China). For Western blot analysis, lysates were separated via SDS‒PAGE and transferred to polyvinylidene fluoride (PVDF) membranes. The membranes were blocked in blocking buffer for 60-100 min, incubated with primary antibodies in dilution buffer overnight at 4 °C, and then incubated with the corresponding secondary antibodies. The protein bands were visualized using an enhanced chemiluminescence (ECL) kit and a chemiluminescence gel imaging system (Vilber, Paris, France). The details of the antibodies used in this assay are listed in Supplementary Table 4.

Co-IP analysis: Cells were washed with cold PBS three times and lysed in lysis buffer for 30 min at 4 °C. The lysates were cleared by centrifugation at 12,000×g (15 min, 4 °C), and the supernatants were transferred to new centrifuge tubes. A BCA Protein Assay Kit was used to determine protein concentrations according to the manufacturer's instructions. Approximately 10% of the supernatant was used for Western blot analysis to measure protein expression, and the remaining lysate was incubated with the indicated antibodies overnight at 4 °C and then mixed with BeyoMag™ Protein A+G beads for 1 h at room temperature. The beads were washed with lysis buffer five times and subsequently boiled in 2×SDS loading buffer for 10 min to collect samples for immunoprecipitation. The details of the antibodies used in this assay are listed in Supplementary Table 4.

### Quantitative RT-PCR (qRT-PCR)

Total RNA was extracted from cells or tissues using TRIzol reagent according to the manufacturer's instructions (Invitrogen, CA, USA). Reverse transcription (RT) was performed with HiScript Q RT SuperMix for qPCR (Vazyme Biotech Co., Ltd, Nanjing, China). RT‒PCR was performed in triplicate with a SYBR Green PCR Kit (Vazyme Biotech Co., Ltd, Nanjing, China) on an Applied Biosystems 7900HT sequence detection system (Applied Biosystems). The primers used are listed in Supplementary Table 2.

### RNA stability assay

For the RNA stability assay, actinomycin D (2 μg/ml; MCE, NJ, USA) was used to inhibit transcription. Cells were collected at the indicated time points after treatment with actinomycin D. Total RNA was subsequently extracted and analysed by qRT-PCR. The level of remaining RNA at each time point was normalized to the level measured at the initial time point (0 h).

### Dot blot assay

The dot blot assay was performed as previously reported 9. Methylene blue (MB) was used to stain mRNAs and as the loading control. The details of the antibodies used in this assay are listed in Supplementary Table 4.

### Proliferation assays

For the CCK-8 assay, one day before treatment, the indicated cells were plated at a density of 1000-2000 cells per well in 96-well plates. After the indicated time, cell viability was determined by using a CCK-8 assay according to the manufacturer's instructions (APExBIO, Houston, USA).

For the clonogenic assay, the indicated cells were seeded in 6-well plates (300-1000 cells per well) and incubated at 37 °C for 10-14 days. Then, the cells were fixed with methanol for 20 min and stained with crystal violet (Beyotime, Shanghai, China) for 30 min.

### Glycolysis assays

For the glucose uptake assay, the indicated cells were first seeded in 6-well plates. After 12 h, the medium was replaced with glucose-free DMEM supplemented with 10% dialyzed FBS. After the indicated time (BGC823 18 h; AGS 6 h), the cells were treated with 2-NBDG (20 μM; Invitrogen) for 1 h, and glucose uptake was quantified via FACS analysis. Lactate production was quantified using a lactic acid assay kit according to the manufacturer's instructions (Jiancheng Bioengineering Institute, Nanjing, China). The extracellular acidification rate (ECAR) was measured with a Seahorse Bioscience XF-24 Extracellular Flux Analyser following the manufacturer's instructions. Briefly, the indicated cells were seeded and cultured in XF24 cell culture microplates (Seahorse Bioscience) for 24 h, and glycolytic activity was assessed using a Seahorse XF Glycolysis Stress Test Kit (Agilent). Sequential injections of various compounds (glucose, oligomycin A and 2-DG) were used to measure glycolytic activity.

### Immunofluorescence (IF) staining

The details of the IF staining protocol were described previously 59. Briefly, the indicated GC cells were incubated first with the indicated antibody at 4 °C overnight and then with the corresponding Alexa Fluor-labelled secondary antibody (Beyotime, Shanghai, China) at a 1:500 dilution for another hour at room temperature. Next, the cells were incubated with DAPI (Beyotime, Shanghai, China) for 5 min. Images of the cells were acquired with a Leica DMi8 system. The antibodies used are listed in Supplementary Table 4.

### RNA Immunoprecipitation (RIP) assay

The RIP assay was performed as previously described 9. Briefly, the MagnaRIP RNA-Binding Protein Immunoprecipitation Kit (Millipore, MA, USA) was used according to the manufacturer's instructions. Lysates of the indicated cells were incubated with beads coated with 5 μg of control IgG (Beyotime, Shanghai, China) or an anti-ac4C antibody with rotation at 4 °C overnight. Next, total RNA was extracted for measurement of HK2 expression by qRT‒PCR. The Primer sequences for acRIP-qPCR are listed in Supplementary Table 2. The antibodies used are listed in Supplementary Table 4.

### Luciferase reporter assay

The indicated cells were seeded in 24-well plates at a density of 6 × 10^4^ cells per well before transfection. AGS NAT10 WT and KO cells were cotransfected with a mixture of luciferase reporter vectors (pGL-SV40-Rluc-TK-Luc) containing the HK2-WT or HK2-Mut sequence (0.8 μg) in to verify the exact site of ac4C modification. After 48 h, luciferase activity was measured using a dual luciferase reporter assay system (Vazyme, Nanjing, China) according to the manufacturer's protocol. The luciferase reporter plasmid was constructed by Corues Biotechnology (Nanjing, China). The HK2-WT (167 nt) sequence used was CCCTCTACAAGCTACATCCTCACTTTGCCAAAGTCATGCATGAGACAGTGAAGGACCTGGCTCCGAAATGTGATGTGTCTTTCCTGCAGTCAGAGGATGGCAGCGGGAAGGGGGCGGCGCTCATCACTGCTGTGGCCTGCCGCATCCGTGAGGCTGGACAGCGATAG; the HK2-Mut1 (167 nt) sequence was CCCTCTACAAG***G***TA***G***AT***G***CT***G***ACTTTGCCAAAGTCATGCATGAGACAGTGAAGGACCTGGCTCCGAAATGTGATGTGTCTTTCCTGCAGTCAGAGGATGGCAGCGGGAAGGGGGCGGCGCTCATCACTGCTGTGGCCTGCCGCATCCGTGAGGCTGGACAGCGATAG; the HK2-Mut2 (167 nt) sequence was CCCTCTACAAGCTACATCCTCACTTTGCCAAAGTCATGCATGAGACAGTGAAGGACCTGGCTCCGAAATGTGATGTGTCTTTCCTGCAGTCAGAGGATGGCAGCGGGAAGGGGGCGGCGCTCATCACTGCTGTGGC***G***TG***G***CG***G***AT***G***CGTGAGGCTGGACAGCGATAG; and the HK2-both Mut (167 nt) sequence was CCCTCTACAAG***G***TA***G***AT***G***CT***G***ACTTTGCCAAAGTCATGCATGAGACAGTGAAGGACCTGGCTCCGAAATGTGATGTGTCTTTCCTGCAGTCAGAGGATGGCAGCGGGAAGGGGGCGGCGCTCATCACTGCTGTGGC***G***TG***G***CG***G***AT***G***CGTGAGGCTGGACAGCGATAG.

### Statistical analysis

All the statistical analyses were performed with SPSS 18.0 or GraphPad Prism 8 software. The significance of differences in the IRSs for NAT10 and HK2 staining between primary tumor tissues and the corresponding normal tissues were assessed by the Wilcoxon test (grouped data). The significance of differences in OS was ascertained by the Kaplan‒Meier method with the log-rank test. Univariate and multivariate Cox regression analyses were used to estimate hazard ratios (HRs) and the associated 95% confidence intervals (CIs), respectively. Then, we analysed the predictive value of parameters using time-dependent receiver operating characteristic (ROC) curve analysis for censored data and calculated the AUCs of the ROC curves as previously reported 59, 60. Representative data are shown as the means ± SDs. Two-tailed unpaired Student's t test was used for comparisons between two groups. One-way ANOVA followed by Tukey's multiple comparisons test was used for comparisons among more than two groups. P < 0.05 was considered to indicate statistical significance. * P < 0.05; ** P < 0.01; *** P < 0.001; P ≥ 0.05, nonsignificant (NS. The experimental data in this study were reliably reproduced in at least three independent experiments or with multiple biologically independent replicates.

## Supplementary Material

Supplementary figures and tables.
