# Supplementary material for: Glucose homeostasis controls N-acetyltransferase 10-mediated ac4C modification of HK2 to drive gastric tumorigenesis
Source: Theranostics. 2025 Jan 20;15(6):2428–50. doi: 10.7150/thno.104310 (PMC11840738; doi:10.7150/thno.104310)
Supplement: Supplementary file 1 — Supplementary figures and tables. [file thnov15p2428s1.pdf]

Supplementary Material

Glucose homeostasis controls N-acetyltransferase 10-mediated ac4C modification of HK2 to drive gastric tumorigenesis

Qiang Wang, Mengmeng Li, Chen Chen, Lei Xu, Yao Fu, Jiawen Xu, Chuanjun Shu, Bo Wang, Zhangding Wang, Changyu Chen, Tao Song, Shouyu Wang

This PDF file includes:

Figure S1. Glucose status controls NAT10-mediated ac4C modification.

Figure S2. NAT10 expression was increased in GC tissues.

Figure S3. NAT10 promotes glucose uptake and lactate release in GC.

Figure S4. NAT10 promotes gastric tumorigenesis.

Figure S5. NAT10 positively regulates HK2 expression.

Figure S6. NAT10 accelerates malignant progression of GC by upregulating HK2.

Figure S7. Targeting the NAT10-HK2 axis in GC cells has clinical value.

Table S1. Agents, inhibitors or agonists.

Table S2. The Oligonucleotides used in this study.

Table S3. The sequences of siRNAs and sgRNAs.

Table S4. Antibodys for Western blot (WB), RIP-qPCR, IF, Co-IP, Dot blot (DB) and IHC.

Supplementary Figures and Figure Legends

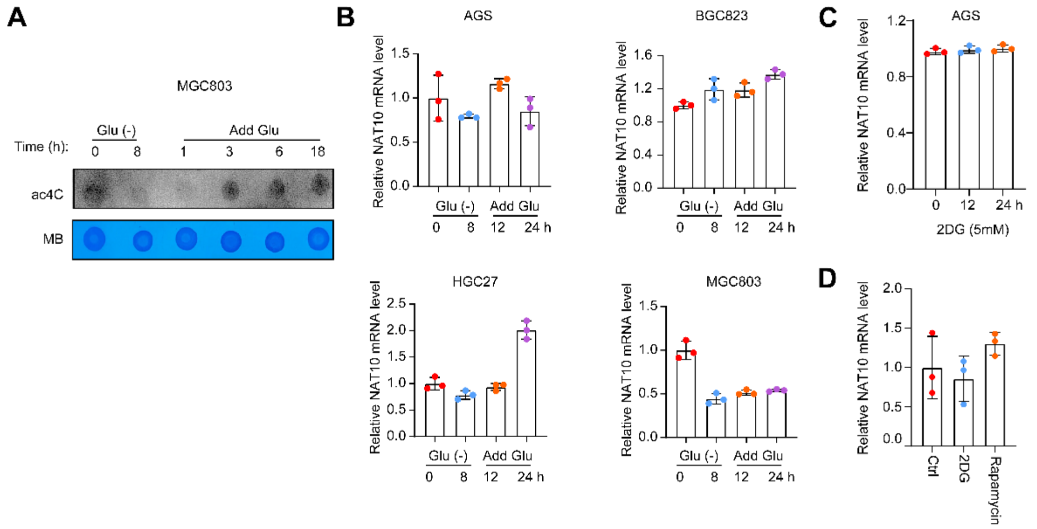

Figure S1. Glucose status controls NAT10-mediated ac4C modification. **(A)** mRNA was isolated from MGC803 cells, which were subjected to glucose starvation for the indicated intervals and then stimulated with glucose (25 mM) for the indicated time. The isolated mRNA was subsequently subjected to dot blot analysis with the indicated anti-ac4C antibody, and MB (methylene blue) staining served as the loading control. **(B, C)** Different GC cell lines (AGS, BGC823, HGC27, and MGC803) were subjected to glucose starvation for the indicated intervals and then stimulated with glucose (25 mM) for the indicated intervals **(B)**; alternatively, AGS cells were treated with 5 mM 2DG for 0, 12 and 24 h **(C)**. The level of NAT10 expression in cell lysates obtained at each time point were measured by qRT–PCR. **(D)** The mRNA level of NAT10 in xenograft tumor tissues from Ctrl group, 2DG treatment group, or Rapamycin treatment group were measured by qRT–PCR (n = 3 tumors). The statistical data in this figure are presented as the mean value ± SD of three independent experiments. Statistical significance was determined by a two-tailed t test. * $P < 0.05$; ** $P < 0.01$; *** $P < 0.001$.

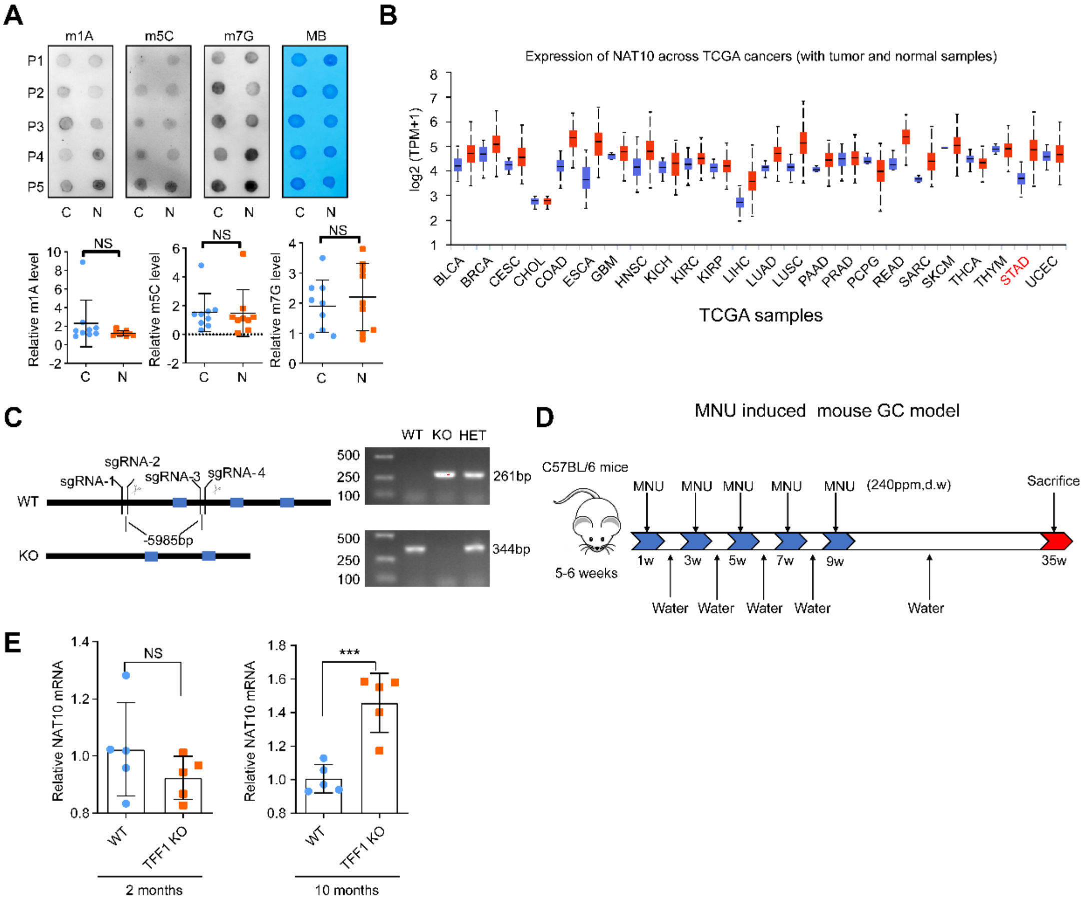

Figure S2. NAT10 expression was increased in GC tissues.

(A) mRNA isolated from GC tissues and paired normal gastric mucosa was subjected to dot blot analysis with different antibodies (specific for m1A, m5C, and m7G), and MB staining served as the loading control (representative images in the upper panel). The relative m1A, m5C, and m7G abundances on mRNA in GC tissues and paired normal gastric mucosal tissues were calculated (bottom panel, n = 9). **(B)** Comparison of NAT10 mRNA expression between tumor and peritumoral tissues in 24 cancer types in the TCGA database. The sample sizes are indicated in the source data. **(C)** Schematic representation of the targeting construct generated using the CRISPR–Cas9 system and used to obtain TFF1-KO mice; sgRNA, single guide RNA; bp, base pairs (left panel); agarose gel electrophoresis of PCR products from WT, KO, and HET mice (right panel). **(D)** Graphical illustration of MNU-induced gastric tumorigenesis in mice. **(E)**The NAT10 mRNA level in the stomach in WT and KO mice at the indicated

time point was measured by qRT–PCR (n = 5). The statistical data in this figure are presented as the mean value ± SD of three independent experiments. Statistical significance was determined by a two-tailed t test. * P < 0.05; ** P < 0.01; *** P < 0.001.

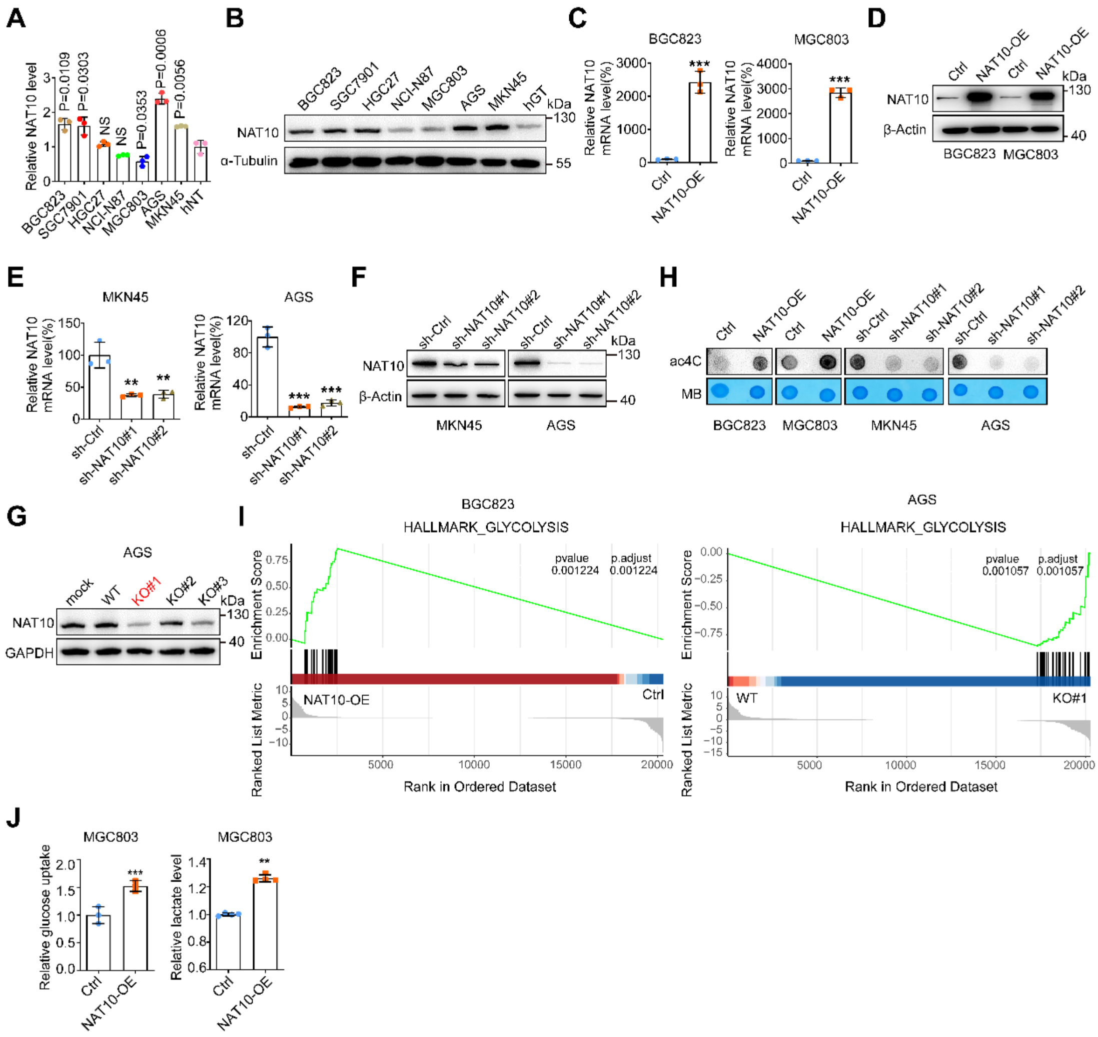

Figure S3. NAT10 promotes glucose uptake and lactate release in GC.

(A) The NAT10 mRNA level was measured in human gastric mucosa tissue (hGT) and different GC cell lines by qRT–PCR. **(B)** The NAT10 protein level was measured in human gastric mucosa tissue (hGT) and different GC cell lines by western blot analysis. **(C)** The overexpression efficiency of NAT10 was verified at the transcriptional level in different GC cell lines by qRT–PCR. **(D)** The overexpression efficiency of NAT10 was verified at the translational level

in different GC cell lines by western blot analysis. **(E)** The knockdown efficiency of NAT10 was verified at the transcriptional level in different GC cell lines by qRT–PCR. **(F)** The knockdown efficiency of NAT10 was verified at the translational level in different GC cell lines by western blot analysis. **(G)** The knockout efficiency of NAT10 was verified at the translational level using different sgRNAs targeting NAT10 in AGS cells by western blot analysis. **(H)** mRNA isolated from NAT10-overexpressing, NAT10-knockdown and the corresponding control GC cells was subjected to dot blot analysis with an anti-ac4C antibody. MB staining served as a loading control. **(I)** GSEA indicated that glycolysis is involved in the alterations in GC progression upon overexpression or knockdown of NAT10. **(J)** Glucose uptake (left panel) and lactate production (right panel) in NAT10-overexpressing MGC803 cells were measured. The statistical data in this figure are presented as the mean value ± SD of three independent experiments. Statistical significance was determined by a two-tailed t test. * $P < 0.05$; ** $P < 0.01$; *** $P < 0.001$.

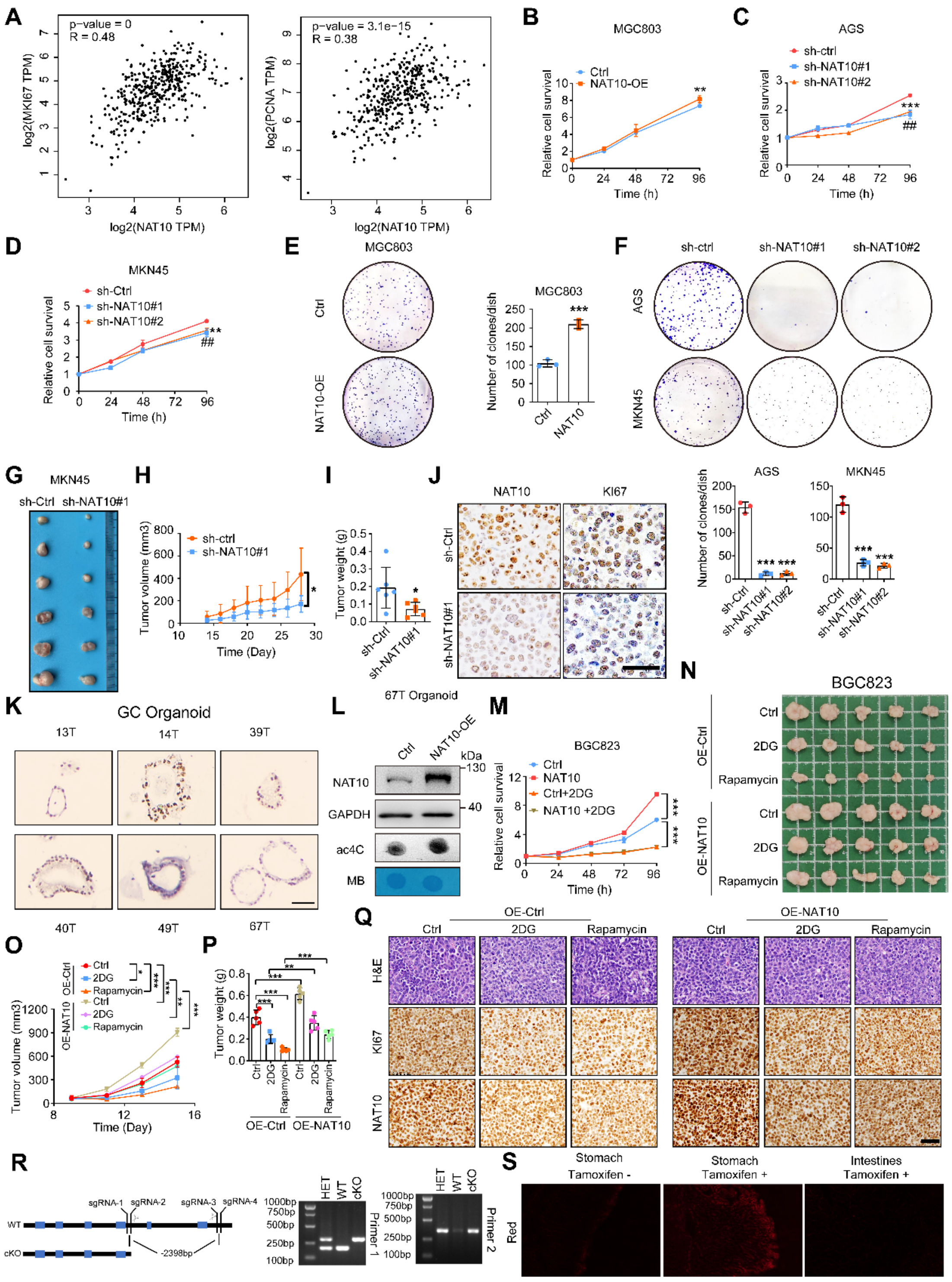

Figure S4. NAT10 promotes gastric tumorigenesis.

(A) NAT10 expression was positively correlated with KI67 and PCNA expression in GC (linear regression), according to analysis with the online bioinformatics tool GEPIA (http://gepia.cancer-pku.cn/). **(B)** A CCK8 assay was used to evaluate the proliferation of MGC803 cells overexpressing NAT10 and

the corresponding control cells. **(C)** A CCK8 assay was used to evaluate the proliferation of AGS cells with NAT10 knockdown and the corresponding control cells. **(D)** A CCK8 assay was used to evaluate the proliferation of MKN45 cells with NAT10 knockdown and the corresponding control cells. **(E)** A colony formation assay was used to evaluate the proliferation of MGC803 cells overexpressing NAT10 and the corresponding control cells. Representative images (left panel) and quantitative results (right panel) are shown. **(F)** A colony formation assay was used to evaluate the proliferation of AGS cells with NAT10 knockdown and the corresponding control cells. Representative images (left panel) and quantitative results (right panel) are shown. **(G)** Knockdown of NAT10 effectively promoted subcutaneous gastric tumor growth in nude mice (n = 6). **(H)** The tumor volume was monitored every other day, and tumor growth curves were generated. **(I)** The tumors were extracted and weighed after 28 days. **(J)** Sections of tumors from the NAT10-knockdown and control groups were stained with anti-Ki-67 and anti-NAT10 antibodies for IHC analysis (scale bars = 50 μm). **(K)** Sections of different GC organoids were subjected to IHC staining with an anti-NAT10 antibody (scale bars = 50 μm). **(L)** The protein level of NAT10 in organoids formed from 67T cells overexpressing NAT10 and the corresponding control cells were measured by western blotting (upper panel). mRNA isolated from NAT10-overexpressing 67T GC organoids were subjected to dot blot analysis with an anti-ac4C antibody (bottom panel). MB staining served as a loading control. **(M)** A CCK8 assay was performed on control and NAT10-overexpressing BGC823 cells subsequently treated with 5 mM 2-DG (2-deoxyglucose) at the indicated times. **(N)** A subcutaneous xenograft model using BGC823 cells overexpressing NAT10 and corresponding control cells was constructed, and these mice were injected with 2DG (800 mg/kg) and Rapamycin (4 mg/kg) intraperitoneally once a day (4 times in total) when the tumor grows to 50-100mm^3 at Day 9. At Day 16, the mice were sacrificed, and the tumors were weighed and imaged (n = 5). The tumor volume was monitored

every other day, and tumor growth curves were generated **(O).** The tumors were extracted and weighed after 16 days **(P).** **(Q)** Sections of tumors were for H&E staining or stained with anti-Ki-67 or anti-NAT10 antibodies for IHC analysis (scale bars = 50 μm). **(R)** Schematic representation of the targeting construct generated using the CRISPR–Cas9 system and used to obtain NAT10 cKO mice; sgRNA, single guide RNA; bp, base pairs (left panel); agarose gel electrophoresis of PCR products from WT, cKO, and HET mice (right panel). **(S)** IF staining was performed on stomach and intestinal tissues from mice treated with or without tamoxifen. The statistical data in this figure are presented as the mean value ± SD of three independent experiments. The *represents statistical analysis between sh-Ctrl and sh-NAT10 #1; $^{\#}$represents statistical analysis between sh-Ctrl and sh-NAT10 #2 in (C and D). Statistical significance was determined by a two-tailed t test. * $P < 0.05$; ** $P < 0.01$; *** $P < 0.001$; $^{\#}$ $P < 0.05$; $^{\#\#}$ $P < 0.01$; $^{\#\#\#}$$P < 0.001$.

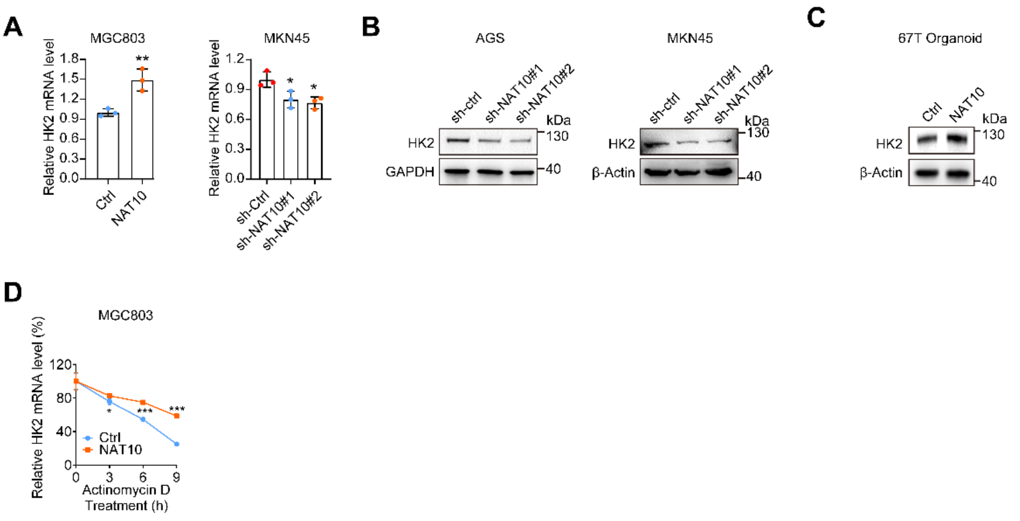

Figure S5. NAT10 positively regulates HK2 expression.

(A) The mRNA level of HK2 in NAT10-overexpressing and NAT10-knockdown GC cells was measured by qRT–PCR. **(B)** The protein level of HK2 in NAT10-knockdown GC cells was measured by western blotting. **(C)** The protein level of HK2 in NAT10-overexpressing 67T organoids was measured by western blotting. **(D)** The level of HK2 expression in NAT10-overexpressing and the

corresponding control MGC803 cells treated with actinomycin D (2 μg/mL) at the indicated time points was measured by qRT–PCR. The statistical data in this figure are presented as the mean value ± SD of three independent experiments. Statistical significance was determined by a two-tailed t test. * P < 0.05; ** P < 0.01; *** P < 0.001.

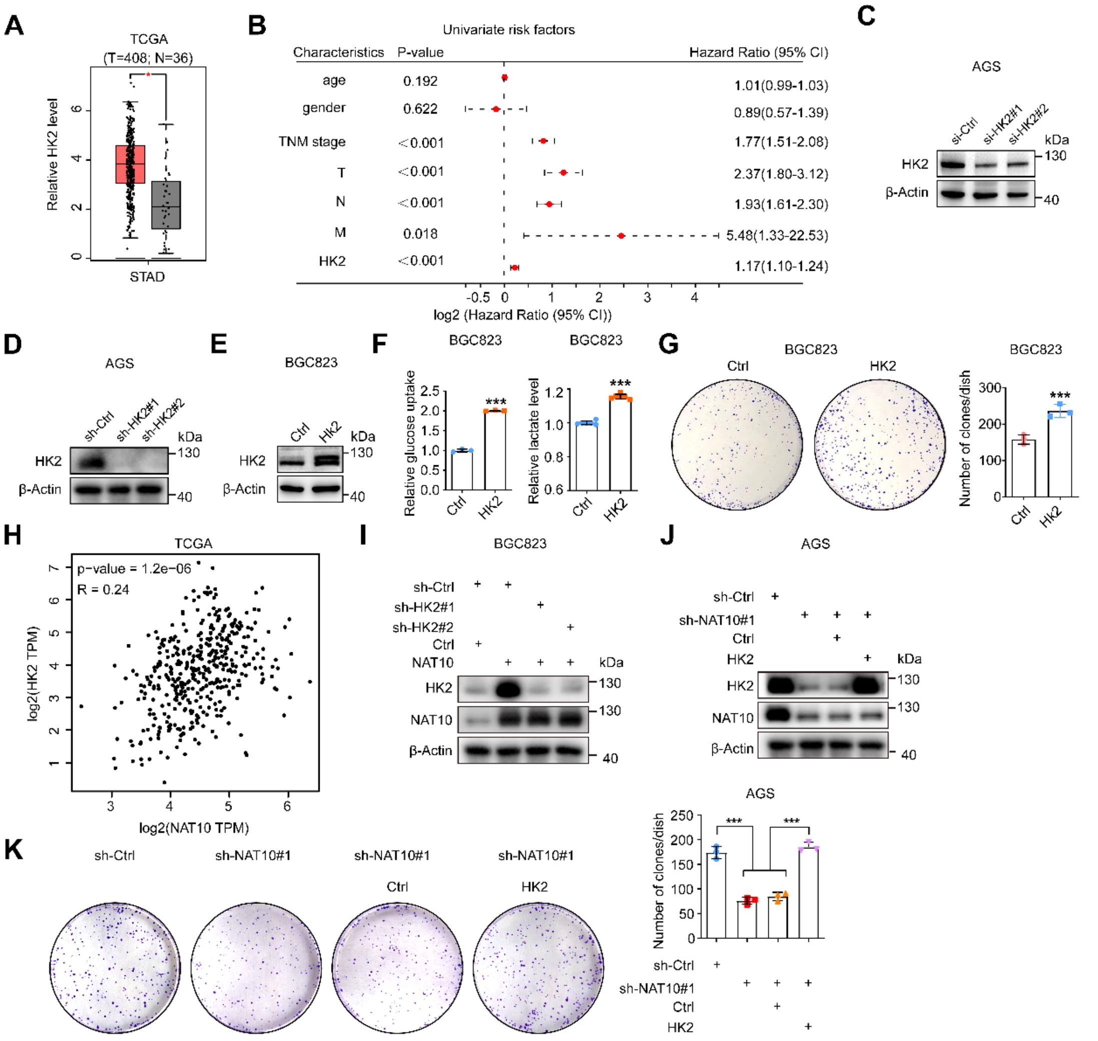

Figure S6. NAT10 accelerates malignant progression of GC by upregulating HK2.

(A) The levels of HK2 expression were analysed in GC (n = 408) and normal gastric mucosal tissues (n = 36) using TCGA data. **(B)** Univariate analyses were performed for the GC cohort. **(C)** The efficiency of HK2 knockdown by its specific siRNAs was verified in AGS cells by western blot analysis. **(D)** The

stable knockdown efficiency of NAT10 by its specific shRNAs lentivirus was verified in AGS cell lines by western blot analysis. **(E)** The stable overexpression efficiency of HK2 was verified in BGC823 cell lines by western blot analysis. **(F)** Glucose uptake (left panel) and lactate production (right panel) in HK2-overexpressing BGC823 cells were measured. **(G)** A colony formation assay was used to evaluate the proliferation of BGC823 cells overexpressing NAT10 and the corresponding control cells. Representative images (left panel) and quantitative results (right panel) are shown. **(H)** NAT10 expression was positively correlated with HK2 expression in GC (linear regression), according to analysis with the online bioinformatics tool GEPIA (http://gepia.cancer-pku.cn/). **(I)** The stable efficiency of HK2 knockdown using its specific shRNAs lentivirus in NAT10-overexpressing BGC823 cells was evaluated by western blot analysis. **(J)** The stable overexpression efficiency of HK2 using its plasmid lentivirus in NAT10 knockdown AGS cells was evaluated by western blot analysis. **(K)** The colony formation ability was evaluated in NAT10-downexpressing AGS cells with or without stable overexpression of HK2 or the corresponding controls. Representative images (left panel) and quantitative results (right panel) are shown. The statistical data in this figure are presented as the mean value ± SD of three independent experiments. Statistical significance was determined by a two-tailed t test. * $P < 0.05$; ** $P < 0.01$; *** $P < 0.001$.

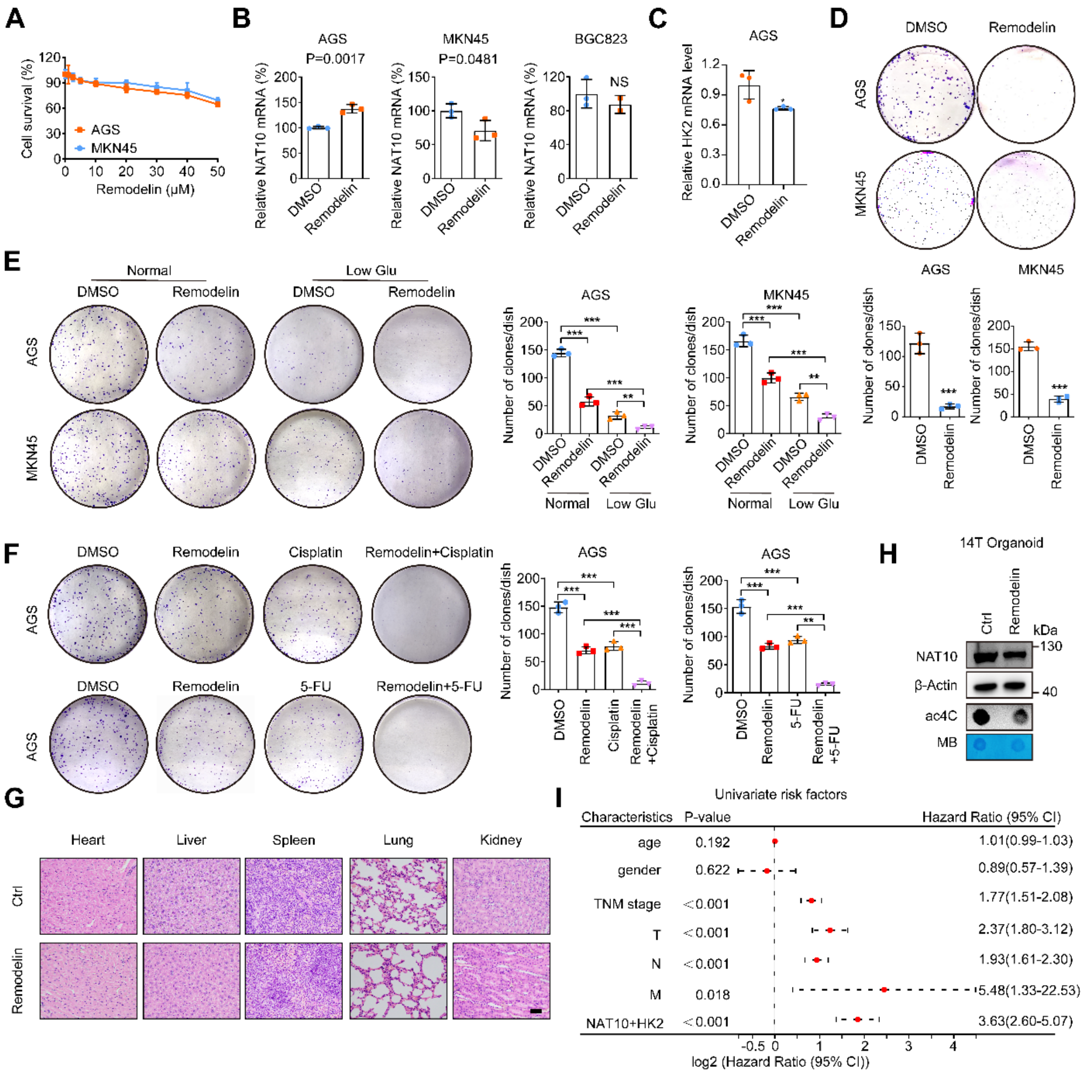

Figure S7. Targeting the NAT10-HK2 axis in GC cells has clinical value.
(A) Different GC cell lines were treated with remodelin at the indicated concentration for 24 h. A CCK8 assay was subsequently used to evaluate cell survival. **(B)** Different GC cell lines were treated with 10 μM remodelin for 24 h. Then, the mRNA level of NAT10 was measured by qRT–PCR. **(C)** AGS cells were treated with 10 μM remodelin for 24 h. Then, the mRNA level of HK2 was measured by qRT–PCR. **(D)** A colony formation assay was used to evaluate the proliferation of different GC cell lines treated with or without 5 μM remodelin. Representative images (left panel) and quantitative results (right panel) are shown. **(E)** A colony formation assay was used to evaluate the proliferation of different GC cell lines treated with or without 5 μM Remodelin in normal cultivation conditions (Glucose 2 g/L) or low glucose (Glucose 0.2 g/L)

cultivation conditions. Representative images (left panel) and quantitative results (right panel) are shown. **(F)** A colony formation assay was used to evaluate the efficacy of Remodelin (5 μM) in combination with common chemotherapy drugs (cisplatin, 0.2 μg/ml; 5-FU, 2 μg/ml). Representative images (left panel) and quantitative results (right panel) are shown. **(G)** Representative H&E staining of major organs, including the heart, liver, spleen, lungs, and kidneys, from mice treated with or without remodelin at the end of the experiment. **(H)** The protein level of NAT10 in 14T organoids treated with or without 10 μM remodelin was measured by western blotting (upper panel). mRNA isolated from 14T organoids treated with or without remodelin was subjected to dot blot analysis with an anti-ac4C antibody (bottom panel). MB staining served as a loading control. **(I)** Univariate analyses were performed for the GC cohort. The statistical data in this figure are presented as the mean value ± SD of three independent experiments. Statistical significance was determined by a two-tailed t test. * $P < 0.05$; ** $P < 0.01$; *** $P < 0.001$.

Supplementary Tables

Table S1. Agents, inhibitors or agonists.

chemicals	Source	Identifier
Glucose (Glu)	MCE	Cat# HY-B0389
2-DG	MCE	Cat# HY-13966
MG132	Selleck	Cat# S2619
PS341	Selleck	Cat# S1013
Leupeptin	MCE	Cat# HY-18234
HCQ	MCE	Cat# HY-B1370
Baf A1	MCE	Cat# HY-100558
Rapamycin	MCE	Cat# HY-10219
MNU	TRC	Cat# CDDY-M325815
Actinomycin D	MCE	Cat# HY-13918
Remodelin	MCE	Cat# HY-16706
Tamoxifen	MCE	Cat# HY-13757A
DharmaFECT4	Dharmacon	Cat# T-2004-03
Lipofectamine 3000	Invitrogen	Cat# L3000015
Puromycin	InvivoGen	Cat# ant-pr-1
Glucose-free DMEM	Gibco	Cat# 11966-025
2-NBDG	Invitrogen	Cat# N13195
Seahorse assay kit	Agilent	Cat# 103020-100
Cisplatin	MCE	Cat# HY-17394
5-Fluorouracil	MCE	Cat# HY-90006

Table S2. The Oligonucleotides used in this study.

**Primer sequences for qRTPCR**	
**Names**	**Sequences (5'-3')**
NAT10 F[a]	GGGATTGGCCTGCAGCATA
NAT10 R[b]	GGCTCCATGACCACATCCTT
GAPDH F	CATGTGGGCCATGAGGTCCACCAC
GAPDH R	GGGAAGCTCACTGGCATGGCCTTCC
HK2 F	TGCCACCAGACTAAACTAGACG
HK2 R	CCCGTGCCCACAATGAGAC
**Primer sequences for PCR genotyping of mice**	
**Names**	**Sequences (5'-3')**
TFF1① F1	TATCTGTTGGTCTGGCCTCAGAAAC
TFF1① R1	TCAGCCTGGAAACTTCCCTGTTC
TFF1② F2	TCTCAGACGAACCTCGTGGTTTG
TFF1② F2	GGCCTACAGCAGCTTTCAGAAGTC
Wild type: ①PCR reaction obtains a single WT band; ②PCR reaction obtains a single WT band. Heterozygote: ①PCR reaction obtains a WT band and a KO band; ②PCR reaction obtains a WT band. Homozygote: ①PCR reaction obtains a single KO band; ② PCR reaction without product. ①WT:6260bp Targeted:~261bp; ② WT:344bp Targeted:0bp	
NAT10① F1	TTCACACAGGAAGTAGAGGCCCAC
NAT10① R1	TTAAAGATGGGAAACAGGGGCAG
NAT10② F2	TGGTCTGCATGGCAGAAAGTC
NAT10② F2	CAGTAAGGAAATCTGCACACAGTTCC
Wild type: ①PCR reaction obtains a single WT band; ②PCR reaction obtains a single WT band. Heterozygote: ①PCR reaction obtains a WT band and a Targeted band; ②PCR reaction obtains a WT band and a Targeted band. Homozygote: ①PCR reaction obtains a single Targeted band; ②PCR reaction obtains a single Targeted band. Note: The sizes of WT and Targeted band are shown below. WT:269bp Targeted:373bp; WT:308bp Targeted:409bp	

Primer sequences for acRIP-qPCR	
**Names**	**Sequences (5'-3')**
HK2 F	CCTCTACAAGCTACATCCTC
HK2 R	GGTTCTATCGCTGTCCAG

a: F, Forward; b: R, Reverse.

Table S3. The sequences of siRNAs and sgRNAs.

**siRNAs**	**Sequences (5’-3’)**
NAT10 siRNA#1	GTACTCCAATATCTTTGTT
NAT10 siRNA#2	GGATGTGCATTCCAGGTAC
HK2 siRNA#1	CTGTGAAGTTGGCCTCATT
HK2 siRNA#2	ACGACAGCATCATTGTTAA
**sgRNAs**	**Sequences (5’-3’)**
NAT10 sgRNA#1	CTCCCACGTTGCCACCATGG
NAT10 sgRNA#2	GGAGTTGAAGGAGAGCTTGC
NAT10 sgRNA#3	CTGCTGTAAGACTCTAGACC

Table S4. Antibodys for Western blot (WB), RIP-qPCR, IF, Co-IP, Dot blot (DB) and IHC.

Antibodies	Source	Identifier
anti-m7G (for DB)	MBL	Cat# RN017M
anti-m6A (for DB)	Abcam	Cat# ab284130
anti-m5C (for DB)	MBL	Cat# D346-3
anti-m1A (for DB)	MBL	Cat# D345-3
anti-ac4C (for DB, IF, and RIP-qPCR)	Abcam	Cat# ab253039
anti-Rabbit IgG (for RIP and Co-IP)	Beyotime	Cat# A7016
anti-Mouse IgG (for Co-IP)	Beyotime	Cat# A7028
anti-HRP mouse (for WB)	Beyotime	Cat# A0216
anti-HRP rabbit (for WB)	Beyotime	Cat# A0208
anti-IHC rabbit (for IHC)	Servicebio	Cat# G1215
anti-IHC mouse (for IHC)	Servicebio	Cat# G1216
anti-β-Actin (for WB)	Beyotime	Cat# AF0003
anti-NAT10 (for WB, IHC, and IF)	Abcam	Cat# ab194297
anti-LC3 (for WB)	Sigma -Aldrich	Cat# L8918
anti-GAPDH (for WB)	Beyotime	Cat# AF0006
anti-SQSTM1 (for WB)	Proteintech	Cat# 18420-1-AP
anti-FLAG (for WB and Co-IP)	Sigma -Aldrich	Cat# F1804
anti-Lamp1 (for IF)	Beyotime	Cat# AG2482
anti-α-Tubulin (for WB)	Beyotime	Cat# AG0126
anti-Ki-67 (for IHC)	Servicebio	Cat# GB121141
anti-HK2 (for WB and IHC)	Proteintech	Cat# 66974-1-Ig
